# Cardiovascular Symptom Tracking Among Patients With Cancer in Cardio-Oncology Care: Qualitative Study Using the Capability, Opportunity, Motivation–Behavior (COM-B) Framework

**DOI:** 10.2196/100279

**Published:** 2026-07-23

**Authors:** Roberto M Benzo, Macy K Tetrick, Rujul Singh, Sara M St George, Vanina Pavia Aubry, Peter Washington, Anvitha Gogineni, Sanam M Ghazi, Olivia Preston, Daniel Addison

**Affiliations:** 1 Division of Cancer Prevention and Control, Department of Internal Medicine, College of Medicine, 3650 Olentangy River Rd, Columbus, OH, United States, 1 614-366-4641; 2 The Ohio State University Wexner Medical Center, The Ohio State University Comprehensive Cancer Center, Columbus, OH, United States; 3 Grossman School of Medicine, New York University, New York, NY, United States; 4 University of Miami Miller School of Medicine, Sylvester Comprehensive Cancer Center, Miami, FL, United States; 5 Department of Public Health Sciences, University of Miami Miller School of Medicine, Miami, FL, United States; 6 Division of Clinical Informatics and Digital Transformation (DoC-IT), Department of Medicine, University of California, San Francisco, San Francisco, CA, United States; 7 College of Medicine, The Ohio State University, Columbus, OH, United States; 8 Cardio-Oncology Program, Division of Cardiology, The Ohio State University Medical Center, Columbus, OH, United States; 9 Cardio-Oncology Program, Division of Cardiology, The University of Texas Southwestern Medical Center, Dallas, TX, United States

**Keywords:** cardio-oncology, symptom monitoring, cancer survivorship, mobile health, digital health, qualitative research

## Abstract

**Background:**

Cardiovascular disease is a leading noncancer cause of morbidity and mortality among individuals diagnosed with cancer. Although modern cancer therapies have improved survival, many are associated with cardiotoxic effects that increase the risk of cardiovascular complications. Early identification of cardiovascular symptoms and signs may support timely clinical management. However, tracking cardiovascular health during cancer care can be complex for both patients and medical team participants, and important indicators, such as blood pressure changes, heart rate variability, fluid retention, chest pain, or new-onset fatigue, may be missed between clinic visits. Mobile health technologies offer potential tools to facilitate remote monitoring, yet behavioral factors influencing cardiovascular tracking in cardio-oncology remain insufficiently understood.

**Objective:**

This study aimed to identify barriers and facilitators influencing cardiovascular symptom tracking among patients with cancer and medical team participants using the capability, opportunity, motivation–behavior (COM-B) framework.

**Methods:**

This qualitative descriptive study included adult patients with cancer (n=12) receiving cardiotoxic therapies and members of their health care teams (n=12), including oncologists, nurses, advanced practice providers, and allied health professionals. Participants were recruited from a cardio-oncology clinic. Semistructured interviews explored perceptions of tracking cardiovascular symptoms and signs. Data were analyzed using rapid qualitative analysis and organized according to COM-B domains. The study received ethical approval, and participants provided informed consent.

**Results:**

Patients with cancer and medical team participants described cardiovascular symptom tracking as a shared responsibility. Capability-related themes included uncertainty regarding which symptoms to monitor, appropriate thresholds for concern, and variability in communication practices. Opportunity-related themes included time pressures, competing clinical demands, and the influence of patient-medical team participant relationships and social support. Motivation-related themes reflected perceived benefits of tracking, including reassurance for patients and improved clinical insight for medical team participants, alongside concerns about information burden and unclear actionability. Both groups expressed interest in digital tools that provide clear guidance, defined response pathways, and integration within health care workflows.

**Conclusions:**

Cardiovascular symptom tracking in cancer care is shaped by interrelated behavioral and contextual factors affecting both patients and medical team participants. Digital systems intended to support tracking should address knowledge clarity, workflow integration, and actionability of reported information. Understanding these determinants may inform the design and implementation of cardiovascular monitoring strategies in cardio-oncology settings.

## Introduction

Cardiovascular disease (CVD) is a leading noncancer cause of mortality in populations with cancer and an important contributor to morbidity. A recent systematic review and meta-analysis of 136 studies across 16 countries reported that individuals with cancer experience a 55% higher risk of cardiovascular mortality compared with the general population (standardized mortality ratio=1.55, 95% CI 1.40‐1.72), with increased risk observed for both heart disease and cerebrovascular conditions [1]. Similarly, a study in the United States shows that patients with cancer remain at increased risk of CVD-related death from the time of diagnosis forward into survivorship, where risk is particularly elevated during the first year following diagnosis [2]. Contemporary cardio-oncology reviews further emphasize that cardiovascular and oncologic conditions are biologically and clinically interconnected, underscoring the need for systematic attention to cardiovascular risk throughout the cancer continuum [3]. Collectively, these findings support the concern for CVD risk in survivorship rather than as a secondary complication in cancer care.

Beyond mortality differences, new cardiovascular events are common during cancer treatment. In a cohort of 17,389 Australian patients treated with chemotherapy, nearly half developed CVD requiring hospital admission during follow-up [4]. More than 50% of these events occurred within 12 months of starting treatment. Patients who developed CVD during follow-up had higher subsequent all-cause mortality [4]. In a population-based cohort study conducted in Alberta, Canada, more than 4.5 million adults were followed for a median of 11.8 (IQR 6.4-11.8) years [5]. Among 224,016 individuals with a new cancer diagnosis, adjusted analyses showed a higher risk of cardiovascular mortality, stroke, heart failure, and pulmonary embolism compared with participants without cancer, while myocardial infarction risk was not increased. Together, these findings suggest that cardiovascular complications frequently arise early in the cancer trajectory and are strongly linked to adverse outcomes, underscoring the need for proactive cardiovascular risk identification and monitoring during and after cancer therapy.

Despite the high incidence of cardiovascular events during and after cancer treatment, early cardiovascular symptoms, such as dyspnea, fatigue, palpitations, or peripheral edema, may emerge between scheduled oncology encounters and may be missed or underdetected [6]. Because these symptoms overlap with common cancer- or treatment-related effects, they may not always prompt cardiovascular evaluation. Systematic electronic monitoring of patient-reported outcomes (PROs) has been linked to positive outcomes in oncology settings. In the multicenter patient-reported outcomes to enhance cancer treatment (PRO-TECT) cluster-randomized trial conducted across 52 community oncology practices, weekly electronic symptom reporting among patients with metastatic cancer significantly reduced emergency department visits and delayed deterioration in physical function, symptom burden, and health-related quality of life (HRQoL) compared with usual care [7]. Similarly, implementation of electronic medical record (EMR)–integrated symptom and needs monitoring in a large ambulatory oncology population at an academic cancer center was associated with reduced emergency department usage and hospitalizations [8]. These findings highlight that systematic symptom tracking can enhance early detection and improve patient-centered and usage outcomes, yet cardiovascular-specific symptom monitoring has not been well-studied in or implemented in cardio-oncology practice.

Digital PRO monitoring and mobile health (mHealth) tools may help address gaps in cardiovascular risk and symptom tracking across the cancer continuum by extending monitoring beyond episodic clinic visits. In a systematic review of mHealth technologies used to study cardiovascular health in populations with cancer, the most commonly captured measures were physical activity and heart rate, primarily via mobile apps and commercial wearables, and integration with EMRs was limited, representing a significant implementation barrier to translating patient-generated data into clinical action [9]. Complementing this landscape, EMR-integrated symptom and needs monitoring in ambulatory oncology has demonstrated that workflow-embedded PRO systems are feasible and associated with reductions in emergency department visits and hospitalizations, while also revealing variability in patient engagement [8]. Qualitative work focused specifically on symptom-tracking for cardiotoxicity, demonstrating that both patients and medical team participants perceive value in a patient-facing mHealth application to facilitate earlier recognition of treatment-related cardiotoxicity. However, medical team participants emphasized the need for simple design, clear triage and escalation pathways for urgent symptoms, and integration with existing clinical workflows and EMR constraints to ensure a safe and feasible implementation [10]. Together, these findings suggest that although digital tools for symptom monitoring are increasingly available, cardiovascular-specific tracking in cardio-oncology remains underdeveloped and insufficiently integrated into routine practice.

Cardiovascular symptom tracking to support cancer care ultimately depends on the behaviors of both patients and medical team participants. In this context, relevant behaviors include patients actively monitoring and reporting cardiovascular signs and symptoms, and medical team participants promoting, reviewing, and responding to that information within routine care. The capability, opportunity, motivation–behavior (COM-B) model, which is central to the Behavior Change Wheel framework, conceptualizes behavior as arising from the interaction of capability, opportunity, and motivation [11]. Capability refers to an individual’s ability to engage in a behavior and includes both physical skills and psychological capacity (eg, knowledge and understanding). Opportunity refers to external factors that enable or constrain behavior, including physical opportunity (eg, time and resources) and social opportunity (eg, support and norms). Motivation encompasses processes that energize and direct behavior, including reflective processes (eg, intentions and beliefs) and automatic processes (eg, habits and emotional responses).

The framework was developed to identify determinants of behaviors and to inform intervention design and has been widely applied to organize barriers and facilitators in implementation research. Applying COM-B to cardiovascular symptom and sign tracking in the cardio-oncology setting provides a structured approach to understanding determinants of these interrelated patient and medical team participant behaviors. In this study, COM-B was used as an analytic framework to classify barriers and facilitators to cardiovascular symptom tracking.

## Methods

### Study Design

We used a descriptive qualitative design guided by the COM-B model. Qualitative data were collected through semistructured interviews focused on identifying barriers and facilitators to tracking symptoms and signs relevant to cardiovascular health (eg, fatigue, chest pain, palpitations, and blood pressure) in the context of cancer treatment and survivorship. Interviews also explored participants’ perspectives on desirable features of digital health solutions to support cardiovascular symptom monitoring, with these features subsequently mapped to COM-B domains.

The target behavior in this study was tracking cardiovascular symptoms and signs between clinical appointments. This behavior involves complementary roles for patients with cancer and medical team participants. Patients engage in tracking using available tools and strategies and report this information during or between visits. The medical team often inquires about cardiovascular health during appointments and may ask patients to monitor specific signs or symptoms between visits to support clinical care discussions and decisions. Because tracking involves actions and expectations on both sides of the clinical relationship, both patients with cancer and medical team participants were interviewed.

The COM-B framework guided both the development of the interview guides and the analysis of qualitative data. The model conceptualizes behavior as the interaction of capability (physical and psychological), opportunity (physical and social), and motivation (reflective and automatic). In this study, the framework was used to classify participant-reported barriers and facilitators related to tracking behaviors.

### Participants and Recruitment

Medical team participants (n=12) and patients with cancer (n=12) who were currently undergoing or had previously undergone cardiotoxic anticancer therapy as defined by established cardio-oncology guidelines [12] (eg, tyrosine kinase inhibitors, chemotherapy, immune checkpoint inhibitors, and radiation) participated in one-on-one semistructured interviews to discuss their experiences and perspectives related to tracking symptoms and signs relevant to cardiovascular health. Eligible medical team participants were (1) aged 18 years or older, (2) English-speaking, (3) currently practicing as a licensed health care professional or allied health professional involved in cancer or cardio-oncology care (eg, physician, advanced practice provider, nurse, or allied health discipline such as physical therapy, exercise physiology, or nutrition), (4) employed within an oncology or cardio-oncology care setting, and (5) involved in the care of patients who were receiving or had received cardiotoxic anticancer therapy.

Eligible patients were (1) aged 18 years or older; (2) English-speaking; (3) diagnosed with cancer; (4) currently undergoing or had previously undergone a cancer regimen that includes a targeted, biologic, radiation, or immune-based therapy (eg, tyrosine kinase inhibitors or immune checkpoint inhibitors); (5) able to engage in physical activity (self-report); and (6) interested in symptom or sign tracking or increasing physical activity levels to improve health (self-report). Although physical activity was discussed during interviews, this analysis focuses on tracking symptoms and signs relevant to cardiovascular health; physical activity–related findings are reported separately.

Medical team participants were recruited through word of mouth and professional referrals. Potential patients were recruited via medical team participant-based referrals within the cardio-oncology clinic. Medical team participants introduced the study to eligible patients during routine medical appointments. If patients expressed interest, members of the research team contacted them via telephone or email to provide additional study information and confirm eligibility. The telephone was the primary mode of contact unless the participant preferred email. If a potential participant could not be reached by phone, a voicemail was left. Research staff followed a 3-call limit protocol when attempting contact. Eligible participants who provided informed consent were enrolled and scheduled for a semistructured interview with a trained member or members of the research team.

Of the 32 patients with cancer screened through the EMR, 7 were ineligible. The remaining 25 preliminarily eligible patients were approached by the clinical study team during medical appointments in the cardio-oncology clinic and asked whether they were interested in speaking with the research study team. The research team attempted to contact all 25 patients by telephone or email. Of the total, 5 patients were unreachable despite repeated contact attempts, and 4 were not contacted after the recruitment target had been met. Moreover, 16 patients were successfully screened by the research team; of these, 2 were ineligible and 2 declined to participate. In total, 12 patients with cancer were enrolled and completed interviews.

### Data Collection

Semistructured interviews were conducted remotely via videoconferencing (eg, Zoom [Zoom Communications] and Microsoft Teams) between December 12, 2023, and July 29, 2024. Interviews were facilitated by the lead author (RMB), one of the research team members (MKT), or both, who were trained in qualitative interviewing techniques and followed a semistructured guide designed to promote open discussion.

The interview guides were developed based on the COM-B model domains, including motivation, opportunity, and capability [11]. Open-ended questions were structured to elicit participants’ perspectives related to tracking symptoms and signs relevant to cardiovascular health. Patients were asked about their experiences tracking their own symptoms and signs, whereas medical team participants were asked about their perspectives on patients’ tracking behaviors. Topics included current tracking practices, contextual factors influencing tracking (eg, available tools and time constraints), motivational factors, and perceptions of potential digital tools to support tracking. Example prompts included: “How do you currently keep track of your own/your patients’ signs and symptoms?” (capability), “Can you describe any opportunities or external factors that help you track signs and symptoms?” (opportunity), and “Can you describe your reasons for tracking signs and symptoms?” (motivation). The full interview guides are provided in Multimedia Appendix 1.

Interviews lasted approximately 60‐90 minutes. All interviews were audio- and video-recorded with the participant’s permission, transcribed using the auto-generated transcript from Zoom or Teams, and reviewed and corrected, when necessary, by a research team member (MKT). Participants were given the option to turn their camera on or off. Participants were assigned unique identifiers not associated with personal identifying information. Participants received an electronic US $25 gift card delivered via email upon completion of the interview.

Sociodemographic information from patients (age, sex, race, ethnicity, education, employment, income, height, and weight) was collected via REDCap surveys. Similar demographic information was collected verbally from medical team participants before interviews, including age, sex, race, ethnicity, job position, and years of experience in their current role.

### Data Analysis

Demographic and medical characteristics were analyzed using descriptive statistics. Rapid qualitative analysis (RQA) was applied to analyze the one-on-one interviews [13-15]. RQA is a team-based qualitative approach in which research team members review audio recordings and transcripts, summarize key points, and identify patterns across interviews to generate themes.

A total of 5 study team members (RMB, MKT, SMSG, VPA, and AG) were involved in transcript review, data summarization, and were adequately trained in RQA. Research assistants first reviewed and corrected auto-generated transcripts for accuracy. Following this step, a researcher (RMB) reviewed each transcript and interview recording and identified segments corresponding to the COM-B domains and subdomains. Relevant excerpts were grouped according to these domains and further categorized as facilitators or barriers.

Each transcript was then independently reviewed by a second researcher (MKT), who repeated the process while having access to the initial coding for comparison. After double review, identified themes and supporting quotes were entered into a structured database to allow for comparison across participants. The research team met regularly to reconcile interpretations and ensure consistency.

Themes were finalized by examining patterns across participants and grouping similar concepts within COM-B subdomains. Because each subdomain is inherently aligned with a COM-B domain, themes were organized accordingly within capability, opportunity, or motivation and illustrated with representative quotes, with subcomponents (eg, physical or psychological capability; physical or social opportunity; and automatic or reflective motivation) used for classification.

### Ethical Considerations

This study was approved by The Ohio State University Institutional Review Board (IRB #2021C0018). All participants provided informed consent before participation. Interviews were audio-recorded with participant permission. Identifying information was removed and replaced with unique identifiers to maintain confidentiality.

## Results

### Participant Characteristics

Participant demographic and clinical characteristics are summarized in Table 1.

**Table 1.** Demographic and clinical characteristics of sample population.

Variables	Patient with cancer (n=12), n (%)	Medical team participant (n=12), n (%)
Average age in years (SD)	65.3 (12.6)	37.8 (6.4)
Average time in position (years)	—^a^	4.5^b^
Sex		
Female	6 (50.0)	7 (58.3)
Male	6 (50.0)	5 (41.7)
Race or ethnicity		
White, non-Hispanic	10 (83.3)	6 (50.0)
White, Hispanic	—	1 (8.3)
Black or African American	1 (8.3)	1 (8.3)
Asian	—	3 (25.0)
Multiple races or ethnicities	1 (8.3)	1 (8.3)
BMI (kg/m^2^)		
18.5‐24.9	3 (25.0)	—
25‐29.9	5 (41.7)	—
≥30	4 (33.3)	—
Average BMI	26.9^b^	—
Education completed		
High school diploma or GED	1 (8.3)	—
More than high school/some college	1 (8.3)	—
College degree	3 (25.0)	—
Graduate or professional degree	7 (58.3)	—
Employment		
Full-time	1 (8.3)	—
Part-time	0 (0)	—
Unemployed	1 (8.3)	—
Retired or disabled	10 (83.3)	—
Annual income (US $)		
50,000-74,999	4 (33.3)	—
75,000-99,999	1 (8.3)	—
100,000-199,999	5 (41.7)	—
Prefer not to answer	2 (16.7)	—
Job position		
Cardio-oncologists	—	4 (33.3)
Oncologists	—	3 (25.0)
Radiation oncologists	—	1 (8.3)
Nurse practitioner	—	1 (8.3)
Physical therapist	—	1 (8.3)
Exercise physiologist	—	1 (8.3)
Dietitian	—	1 (8.3)
Cancer site		
Acute myeloid leukemia	3 (25.0)	—
Breast	2 (16.7)	—
Prostate	1 (8.3)	—
Lymphoma	1 (8.3)	—
Multiple myeloma	1 (8.3)	—
Uterine	1 (8.3)	—
Chronic lymphocytic leukemia	1 (8.3)	—
Lung	1 (8.3)	—
Neuroendocrine malignancy	1 (8.3)	—
Cancer treatment received		
Chemotherapy	6 (50.0)	—
Radiation	5 (41.7)	—
Stem cell transplant	4 (33.3)	—
Tyrosine kinase inhibitors	4 (33.3)	—
Hormonal therapy	3 (25.0)	—
Surgery	3 (25.0)	—
Immune checkpoint inhibitors	1 (8.3)	—
Other	4 (33.3)	—

a Not applicable.

b % value not applicable.

In total, 12 patients with cancer participated in interviews. The mean age was 65.3 (SD 12.6) years, and participants were evenly distributed by sex (6/12, 50% female). Most identified as White and non-Hispanic (10/12, 83.3%). The mean BMI was 26.9 kg/m² (SD 4.53), with 41.7% (5/12) classified as overweight and 33.3% (4/12) classified as obese. The majority had completed a graduate or professional degree (7/12, 58.3%) and were retired or disabled (10/12, 83.3%). The most frequently reported annual household income category was US $100,000-US $199,999, representing 41.7% (5/12) of participants. Cancer diagnoses included acute myeloid leukemia (3/12, 25%), breast cancer (2/12, 16.7%), and a range of other malignancies represented in smaller frequencies. Participants reported receiving multiple cancer-directed therapies, including chemotherapy (6/12, 50%), radiation (5/12, 41.7%), tyrosine kinase inhibitors (4/12, 33.3%), stem cell transplant (4/12, 33.3%), hormonal therapy (3/12, 25%), surgery (3/12, 25%), immune checkpoint inhibitors (1/12, 8.3%), and other therapies (4/12, 33.3%).

A total of 12 medical team members were interviewed. Most participants were female (7/12, 58.3%). The mean age was 37.8 (SD 6.4) years, and participants had been in their current position for a mean of 4.5 (SD 2.78) years. Participants identified as White and non-Hispanic (6/12, 50%), Asian (3/12, 25%), White and Hispanic (1/12, 8.3%), Black or African American (1/12, 8.3%), or multiple races or ethnicities (1/12, 8.3%). Professional roles included cardio-oncologists (4/12, 33.3%), oncologists (3/12, 25%), radiation oncologists (1/12, 8.3%), and allied health professionals, including a nurse practitioner, physical therapist, exercise physiologist, and dietitian (each 1/12, 8.3%).

### Qualitative Themes

Themes were organized by COM-B domain and categorized as facilitators or barriers. Table 2 summarizes the themes identified among patients and medical team participants, along with the number of participants who endorsed each theme. Illustrative quotes can be found in Multimedia Appendix 2.

**Table 2.** Facilitators, barriers, and desired digital health features for cardiovascular symptom tracking, mapped to COM-B^a^ domains.

Domain and subdomain	Theme	Patients, n	Medical team, n
Capability			
Facilitators			
Psychological	Awareness/knowledge of own symptoms/health	12	—^b^
Psychological	Was made aware of cardiotoxicity risks by medical team and asked to track	7	—
Physical	Journaling, using other recording tools to track	7	—
Psychological	Knowledge of cardiotoxicity	—	11
Physical	Medical team participant asks about symptoms	—	12
Barriers			
Psychological	Did not know about cardiotoxic risks or how, when to track	5	—
Psychological	Recall bias	5	—
Psychological	Lack of tech-savviness	4	—
Physical	Symptoms deterred them from tracking	4	—
Psychological	Hard to know which symptoms are cardiotoxic-related	—	8
Opportunity			
Facilitators			
Social	Patient–medical team participant encounters	11	7
Social	Social support	8	7
Physical	Physical and digital tools	11	10
Social	Staff support	—	5
Barriers			
Physical	Lack of time	6	7
Social	Medical team participant not requesting data	6	—
Physical	Lack of physical or digital tools	—	10
Social	Limited staff support	—	2
Motivation			
Facilitators			
Automatic	Habitual tracking	7	3
Reflective	Perceived value of monitoring for health management	11	—
Automatic	Emotional processes	6	—
Reflective	Planning their daily lives	2	—
Reflective	Clinical decision making	—	10
Reflective	Patient motivation and awareness	—	5
Reflective	Understanding health outside of the clinic	—	3
Barriers			
Reflective	Lack of emphasis to track from medical team participants	3	—
Reflective	Overwhelmed by demands of being a survivor	4	—
Automatic	Emotional processes	6	—
Reflective	Don’t want overload of information	—	4
Reflective	Patient motivation and burden	—	5
Digital health solution features			
Capability			
Simple design	—	6	5
Entering and tracking data	—	9	—
Integrate with other devices	—	3	—
Opportunity			
Reminders, prompts, widgets	—	7	—
Reminders, alerts, triaging features	—	—	7
Medical team participant communication	—	4	—
Educational insights	—	6	—
Motivation			
Tracking features	—	4	—
Tracking signs and visualizing trends	—	—	6
Reward system	—	1	4
Motivational messaging	—	3	—
Gamification	—	2	—
Sharing with medical team participant	—	2	—

a COM-B: capability, opportunity, motivation–behavior.

b Not applicable.

Within the capability domain, 3 facilitator themes and 4 barrier themes were identified among patients, compared with 2 facilitator themes and 1 barrier theme among medical team participants. Opportunity included 3 facilitator themes and 2 barrier themes among patients, and 4 facilitator themes and 3 barrier themes among medical team participants. Within the motivation domain, 4 facilitator themes and 3 barrier themes were identified among patients, while 4 facilitator themes and 2 barrier themes were identified among medical team participants.

Digital health solution features were also described and mapped to COM-B domains. Among patients, 5 motivation-related, 3 opportunity-related, and 3 capability-related features were identified. Among medical team participants, 2 motivation-related features and 1 feature related to opportunity and capability were identified.

### Domain: Capability

#### Overview

In the COM-B framework, capability refers to an individual’s capacity to engage in a behavior. Capability includes psychological capability, such as knowledge, cognitive skills, and memory processes, and physical capability, such as the physical ability to perform the behavior. In the context of cardiovascular symptom tracking, capability reflects whether patients with cancer and medical team participants possess the knowledge, skills, and abilities necessary to monitor and interpret signs and symptoms. We identified 3 facilitator themes and 4 barrier themes among patients, and 2 facilitator themes and 1 barrier theme among medical team participants. Patients described varying levels of awareness of cardiotoxicity risks and challenges with real-time documentation, while medical team participants noted difficulty attributing symptoms specifically to cardiotoxicity.

#### Psychological Capability

The first theme related to knowledge of cardiovascular risk. Medical team participants described awareness that certain cancer treatments increase the risk of CVD. In contrast, although patients described awareness of their symptoms, just over half reported being aware that their cancer treatment could increase cardiovascular risk. Some patients reported that they had not been informed about the potential cardiac effects of cancer treatment. As some patients described:

*They tested my heart before I was allowed to have the transplant, but I don’t believe they said anything about the risk to my heart causing any heart problems.*

*I never really thought to ask about well, how will this affect my heart, or will this affect my heart in a bad way, or whatever.*

*No, we didn’t discuss that [increased cardiovascular risk]. I don’t remember. Well, I don’t, I don’t remember them discussing that, but I guess if I had looked at the papers that come with all the drugs that one drug that they took me off of definitely can impact the heart can be a side effect.*

Medical team participants described obtaining information about signs and symptoms during clinical encounters and noted difficulty determining whether symptoms were related to cardiotoxicity, given the variability in patient presentation. As one medical team participant stated:

*They’re not always related to cardiotoxicity, it could just be coincidental…but then, other times, it’s clearly due to their cardiotoxicity of their cancer therapy.*

Most patients reported that their medical team had asked them to track signs and symptoms, and some described already monitoring select measures. However, several patients expressed uncertainty about which information to track and reported challenges documenting symptoms in real time. Some patients also reported relying on memory to recall symptoms between appointments rather than documenting them in real time. Only a subset of patients described using systematic tools, such as journals or applications, to record cardiovascular symptoms over time.

*I don’t know what else would be important to track of the, you know, what’s being tracked. Blood pressure, all this other stuff.*

*You know, [oncologist name] has been asking me, and once again this is my recall. I could not be remembering correctly. But he, you know, he mainly asked, “Do you have chest pain? Do you have this? Do you have that?” But I haven’t been able to like, pull out a notebook and say, “On February third I had this and February second…*”

Finally, some patients noted that their lack of tech-savviness was a barrier to digital tracking. As one patient noted:

*Oh, you gotta, you gotta keep in mind, I'm a bit technologically challenged.*

#### Physical Capability

Physical capability refers to the physical capacity to perform a behavior. In the context of tracking cardiovascular signs and symptoms, this includes whether patients feel physically able to consistently monitor and document symptoms in their daily lives.

Some patients described that severe symptoms, such as fatigue or feeling unwell, limited their ability to consistently track signs and symptoms. When symptoms were most burdensome, tracking was sometimes deprioritized or not completed at all.

### Domain: Opportunity

#### Overview

In the COM-B framework, opportunity refers to external factors that make a behavior possible or prompt it. Opportunity includes both social (interpersonal influences, cultural norms, and professional roles) and physical (environmental resources, tools, time, and system-level structures) opportunities. In the context of cardiovascular symptom tracking, opportunity reflects the extent to which the clinical environment, social networks, and available tools support or constrain tracking behaviors for both patients with cancer and medical team participants. We identified 3 facilitator themes and 2 barrier themes among patients, and 4 facilitator themes and 3 barrier themes among medical team participants. Both groups highlighted patient–medical team participant encounters, social support, and tool access as facilitators, while time constraints and limited standardized resources emerged as key barriers.

#### Social Opportunity

The first theme identified by both patients and medical team participants was the role of patient–medical team encounters in shaping tracking behaviors. Most medical team participants described obtaining information about signs and symptoms during follow-up visits. Similarly, most patients reported that tracking was more likely to occur when their medical team explicitly asked them to monitor specific signs and symptoms between appointments. Conversely, some patients described that when tracking was not requested, they were less likely to engage in monitoring. For example, as 2 participants noted:

*…during treatment for radiation for like esophagus cancer, for example, it’s a five and a half week long treatment. So, we see them every week. They, you know, they kind of report what’s going on every week.*

*…I think my doctors definitely help, obviously. But they they’ve been awesome, and even in terms of explaining like, why, it’s important. I think that’s like a huge thing that sometimes providers miss, is explaining why specifically we’re tracking these things and why it’s important that we’re kind of tracking them for ourselves, what they could in indicate in the future. And then they’ve also been very good at kind of explaining everything, and the process of how to do that.*

Another theme identified by both groups was the role of social support (ie, family, friends, and social support groups) in tracking signs and symptoms. Medical team participants described that family members and friends could contribute additional information about patients’ symptoms and help reinforce monitoring behaviors (eg, medications and blood pressure). For example, 1 medical team participant stated:

*So, their family and friends are huge in getting the full picture of the patients a lot of times… So, getting all the information and, or, tracking like you said, tracking symptoms, making sure they’re taking their medication, making sure they’re taking their blood pressures, not downplaying their blood pressures if there’s something that should be concerned.*

Patients also described family members and friends as providing support for tracking, including encouragement and concern when symptoms occurred. One patient stated:

*My husband has been great cause he wants me to feel good. So, I think, having like a support system of people that want you to feel as good as you can and are concerned when you don’t feel good and kind of want to mark that, that’s important.*

A related theme among medical team participants involved staff roles in communication and follow-up. Participants described nurses as frequently serving as a point of contact between patients and oncologists. Some medical team participants also described workload and staffing constraints that affected their ability to follow up on patients’ signs and symptoms outside of scheduled visits. For example, 1 medical team participant stated:

*I don’t have the bandwidth to call every patient and check on them and the patients I see here on Wednesday. [Nurse’s name] helps with that.*

#### Physical Opportunity

Among both patients and medical team participants, most reported using physical and digital tools to track signs and symptoms. Most patients reported using paper-based tools (eg, notebooks, binders, or written logs). Patients also described using digital tools (eg, note-taking applications, meditation or glucose monitoring apps, blood pressure cuffs, and KardiaMobile) and device features (eg, alarms) to monitor signs and symptoms. A few patients reported not using any applications to track signs or symptoms. Some patients reported that having physical or digital tools organized in a single location helped with tracking. For example, 1 participant noted:

*I found in life that it helps to be a little more organized. You know I mentioned. I try to always have the blood pressure cuffs in a one place, and it’s taken me a long time in life to realize that being organized really helps.*

Most medical team participants described limited availability of paper-based or digital tools to provide to patients for tracking. Participants on the medical team reported lacking standardized resources to monitor patients between visits. For example, 1 medical team participant stated:

*I think leveraging, like, 21st century technology to help patients, like, better identify and capture their symptoms, and then to also help them kind of engage in the preventative, or, you know, meaningful cardiac preservation or survivorship activities is really kind of a big part of the future.*

Another theme identified among both the medical team and patients was the time barrier. Most medical team participants consider time constraints to be a major barrier to understanding trends in patients’ signs and symptoms. Similarly, many patients mentioned that finding time to track their signs and symptoms was a common obstacle.

*So, I think cause sometimes, especially when I was working full time and also trying to like manage treatments and stuff, it just felt like there was such a limited amount of time and memory to do those things [tracking].*

### Domain: Motivation

#### Overview

Motivation within the COM-B model refers to processes that direct and energize behavior. It includes reflective motivation, which involves conscious decision-making, planning, and evaluation of outcomes related to tracking behaviors, and automatic motivation, which involves emotional responses and habitual processes that influence engagement in tracking cardiovascular symptoms and signs. Themes were identified within both reflective and automatic motivation among patients and medical team participants. We identified 4 facilitator themes and 3 barrier themes among patients, and 4 facilitator themes and 2 barrier themes among medical team participants. Patients described reassurance and a sense of control as motivating tracking, while survivorship demands and emotional distress could diminish engagement; medical team participants valued tracking for clinical decision-making but expressed concern about information volume and actionability.

#### Reflective Motivation

Patients identified the perceived value of monitoring for health management and planning. Patients reported recognizing the importance of tracking their cardiovascular symptoms and described this recognition as motivating their engagement in tracking behaviors. Participants reported that monitoring contributed to a clearer understanding of their symptoms and allowed them to observe patterns or changes in symptoms over time. For example, 1 patient stated:

*The one thing that I enjoy is being able to look at the data. I mean, that’s just my thing. I like being able to look and see my trends where I’ve been, where I’m going.*

Another patient shared:

*Honestly, I think that knowledge and data are kind of like a huge piece of mind for me personally like being to look able to look at trends especially like makes me think about chemo both with, you know, the signs of like my blood pressure, my heart rate, but also with the symptoms being able to kind of plan how I’m going to feel, I know on Day 5. I’m going to feel, you know, the most tired, and things like that. Allows me to kind of find the best way to live my life to the max, you know.*

However, some patients reported that when the medical team participants did not emphasize the importance of monitoring, they were less likely to prioritize tracking. Participants described reduced urgency or engagement when tracking was not explicitly discussed during clinical encounters. One patient noted:

*I think that’s like a huge thing that sometimes providers miss, is explaining why specifically we’re tracking these things and why it’s important that we’re kind of tracking them for ourselves, what they could in indicate in the future.*

The second theme identified among patients was the use of tracking signs and symptoms to inform planning of daily activities and life events (eg, travel, holidays, and family events). Patients described using tracked information to guide their activities, adjusting plans based on how they felt or anticipated feeling. One patient described avoiding travel during treatment due to anticipated side effects and stated:

*If I know when I’m going to be tired, I’m not going to make plans for those days. I’m going to try to give myself rest. But then, on the days where I know I start ticking up, then I know that I can start making plans with people, or we can choose to take a trip at that time.*

Some patients also described feeling overwhelmed by the demands of managing their health, which they reported affected their engagement in tracking. One participant shared:

*I think it gets to be just a lot sometimes. Both logistically, in terms of like, there’s so many appointments, there’s so many medications. There are so many points, again, of like data, of like taking care of yourself, that sometimes you’re just like, I want to sit on this couch all day. I don’t want to think about any of this crap, like I need a day or 2 or 3, you know.*

The first theme identified among medical team participants was the use of signs and symptom data to inform clinical decision-making. Medical team participants described using PROs and other tracking data (eg, EMR data) to guide treatment adjustments and medication management. Participants also described tracking data as important for monitoring and identifying changes in cardiovascular health status. For example, 1 medical team participant stated:

*But having better kind of updates on their symptom progression will allow us to better manage their cardiotoxicity or cardiovascular disease that may be independent from their cancer, cancer therapy.*

Medical team participants also described using symptom-tracking data to address specific patient complaints and inform clinical management decisions. Participants noted that tracking data provided additional context when evaluating symptoms. For example, 1 medical team participant stated:

*You ask them whether they’re doing okay, whether they’re having any symptoms. If they’re having any symptoms, then you try to make adjustments to the medications.*

In contrast, some medical team participants described information overload as a potential barrier to encouraging tracking. Participants noted concerns about receiving large volumes of patient-generated data and the time required to review it. For example, 1 medical team participant stated:

*You like run the risk of collecting too much information, and, you know, kind of overburdening… You know, everybody’s busy nowadays. And so, like sorting through you know, like reams of paperwork is not necessarily gonna be super helpful.*

The second theme identified among medical team participants was encouraging tracking to obtain information about patients’ health outside the clinic, in their real-world environments. Participants described that clinical measurements may differ from those recorded at home and that tracking between visits provides additional context about patients’ status. For example, 1 medical team participant stated:

*Usually, I ask them, you know, “When you go home over the next, you know, month or so before I see you next, I want you to write down your blood pressure at different times of the day and make sure that you bring that back. So I know, you know, is your blood pressure really high at home, or is it just high in clinic because you’re anxious?”*

Medical team participants described patient motivation as influencing engagement in tracking, noting variability in how consistently patients monitored and reported symptoms. Participants also described that asking patients to track signs and symptoms could add to patients’ responsibilities, which, in some cases, limited engagement. At the same time, medical team participants reported encouraging patients to track their health between visits and emphasized the importance of patients being aware of changes in their health status. Some participants described prompting patients to take an active role in monitoring their health. For example, 1 medical team participant noted:

*The big thing is for them to be self-aware of what’s going on… So, being more self-sufficient and taking control of their health is the biggest goal, because, like so, we can’t hold their hand forever.*

#### Automatic Motivation

Medical team participants described routinely asking about signs and symptoms during patient encounters, characterizing this practice as part of standard workflow. Similarly, several patients described tracking as part of their daily routine. Patients reported that engaging in routine tracking was reassuring and associated it with feeling more in control of their health. One patient shared:

*It’s just kind of a routine. I guess it just kind of reassures me that I know what’s right. I just feel like it’s probably, you know, a lot psychological, but it feels like I’m keeping track of something, my body, you know, and I’ve got control of something right?*

The second theme among patients revealed that emotional processes can influence the tracking of signs and symptoms. Patients mentioned concerns about their health and the potential for worsening conditions, which may motivate them to monitor their symptoms closely, as they seek to anticipate changes and mitigate negative outcomes.

*I probably subconsciously was more apt to worry about the cancer coming back. I probably worried more about any symptoms or anything and contributing to that.*

In contrast, some patients described that frequent or intense tracking became distressing or anxiety-producing. One participant shared:

*Sometimes I see myself as going overboard with tracking so much that you become too somatically focused, and it starts becoming anxiety-producing. Because you’re always tuned into every breath, and this and that, rather than living your life and having fun and doing things that are fun.*

### Digital Health Solution Features

Both patients and medical team participants described specific features that could support cardiovascular symptom tracking.

Among medical team participants, the most frequently endorsed feature was reminders, alerts, or triaging capabilities (7/12). Medical team participants described the value of automated prompts to encourage patient logging, as well as alert systems that would highlight abnormal values and help prioritize review. One participant noted:

*I will say reminders and things like keeping track of the blood pressure log, etc., through the phone, that works.*

Another medical team participant emphasized the importance of triaging information efficiently:

*... like you only get the red flag if it’s a lab that’s abnormal or it’s an imaging say, that’s abnormal. So, in theory, when you’re going through your inbox, you kind of triage, what’s what I really need to like open up and look at versus it’s probably normal.*

Of the 12 medical team participants, 6 highlighted the importance of tracking functionality that allows visualization of trends over time. Medical team participants described wanting both patients and medical team participants to see symptom progression and recovery patterns:

*I’ve often thought like how nice it would be to, to, you know, kind of keep track of patients when they’re not in my clinic to make sure that everything’s going okay, and they’re not in trouble, but then also to for the patient to kind of log things and kind of see their progress and see whether you know how they’ve progressed and how they’re recovering. So, I think it’d be, you know, valuable in multiple aspects.*

Moreover, 4 medical team participants emphasized the potential benefit of incorporating reward systems or positive reinforcement to promote long-term tracking behaviors. One participant explained:

*If they have some other commendation for whatever they’re done in an app then they’re more likely to do… and that’s supposed to reinforce and something like that would definitely help people start doing or sticking onto the habit.*

Finally, 5 medical team participants stressed that any digital solution must be simple and user-friendly, with clear and actionable outputs:

*I think if the app is simple, I think it could be helpful, that the app is simple and captures relative symptoms that are, that are useful, and that can be turned into actionable items that would be useful.*

## Discussion

### Principal Findings

This qualitative study examined barriers and facilitators to cardiovascular symptom and sign tracking from both patient and medical team perspectives, in a cardio-oncology setting, using the COM-B framework. We found that tracking behaviors related to cardiovascular health were shaped by interrelated factors across capability, opportunity, and motivation domains. Patients described variability in knowledge regarding cardiovascular health during and after cancer treatment, including uncertainty about which symptoms or signs to monitor and when to communicate changes. Medical team participants similarly identified gaps in standardized processes for cardiovascular symptom tracking and expressed concerns about time constraints and information burden. At the same time, both groups identified various motivational drivers, including perceived value of monitoring for maintaining cardiovascular health, reassurance, and improved clinical decision-making. Social opportunity, particularly patient–medical team participant communication and family involvement, also emerged as central to supporting tracking behaviors.

These findings suggest that cardiovascular symptom tracking in cancer care is not limited by awareness of cardiotoxicity alone but is influenced by clarity of expectations, workflow integration, and alignment between patient and medical team participant roles.

### Comparison With Previous Literature

Randomized trials in oncology have demonstrated that structured electronic symptom monitoring can improve some clinical and PROs. For example, in the PRO-TECT cluster-randomized trial, weekly electronic symptom reporting reduced visits to the emergency department and delayed deterioration in physical function and HRQoL among patients with metastatic cancer [7]. Similarly, the use of an EMR-integrated symptom and needs monitoring in an ambulatory, racially, ethnically, and linguistically diverse oncology population (My Wellness Check [American Heart Association]) has been associated with reductions in emergency department usage and hospitalization [8]. These findings are consistent with broader evidence that PRO monitoring enhances symptom detection and clinical communication, supporting improved system-level outcomes in oncology care [16].

These studies establish the potential clinical impact of systematic symptom monitoring but do not examine the behavioral mechanisms influencing long-term engagement in symptom tracking, particularly for cardiovascular risk in cardio-oncology contexts.

Our findings extend this literature by identifying capability-, opportunity-, and motivation-related determinants influencing the adoption and implementation of cardiovascular symptom tracking. Whereas previous trials primarily evaluate downstream clinical outcomes, our qualitative data illuminate upstream behavioral and contextual factors (including knowledge gaps, workflow constraints, uncertainty about symptom attribution, and perceived role boundaries) that may influence the feasibility of structured cardiovascular monitoring.

Our findings align with the qualitative work on cardiotoxicity-focused mHealth tools, where both patients and medical team participants expressed interest in digital cardiovascular symptom tracking but stressed the importance of clear reporting guidance for urgent symptoms, integration with existing clinical systems, and minimal workflow disruption [10]. Additionally, a recent systematic review of mHealth tools for cardiovascular health in patients with cancer found a fragmented array of technologies and measures, limited integration with electronic health records (EHRs), and few studies specifically addressing structured cardiovascular symptom collection [9]. More broadly, mHealth technologies enable continuous monitoring of both physiological and patient-reported data, but their implementation is often constrained by variability in engagement and limited integration into clinical care workflows [9], aligning with recent implementation-focused reviews demonstrating that the effectiveness of remote symptom monitoring depends on patient engagement, workflow integration, and actionable use of reported data [16].

Together, these studies suggest that although digital monitoring tools are increasingly available, implementation may depend less on technological capability alone and more on alignment with behavioral determinants and system-level structures. This aligns with findings from previous work on EHR-integrated interventions, which highlight that successful implementation depends on alignment with clinical workflows, defined roles, and manageable data flows rather than technology alone [17,18]. By applying the COM-B framework, our study provides a behavioral lens to inform the design and integration of cardiovascular symptom-tracking approaches in oncology settings. Notably, cardiovascular symptom tracking may differ from general oncology symptom monitoring in several respects: the overlap between cardiotoxicity-related and cancer- or treatment-related symptoms, the evolving nature of cardiovascular risk across the cancer care continuum, and the need for coordination across oncology and cardiovascular specialties.

### Implications for Behavioral Determinants and Implementation

Cardiovascular symptom tracking in cancer care emerged as a shared behavior shaped by capability, opportunity, and motivation among both patients and medical team participants. Patients were often unsure which cardiovascular symptoms or signs should be tracked and when changes should prompt communication, suggesting that education alone may be insufficient without clear expectations for monitoring and reporting. Although patients generally recognized the relevance of cardiovascular health, many described ambiguity about expectations for monitoring and reporting. Medical team participants similarly described variability in how cardiovascular monitoring was addressed and incorporated into routine care. Such variability likely reflects differences in clinical context, patient complexity, and workflow demands, underscoring the importance of clarifying expectations and responsibilities when considering strategies to support consistent tracking practices. These challenges highlight the importance of clearly defining which cardiovascular symptoms should be tracked and when reporting is warranted, particularly in contexts where symptom attribution is uncertain. They also suggest that effective tracking approaches may need to be integrated into existing workflows and support coordination across oncology and cardiovascular care teams, agreeing with broader systematic reviews identifying knowledge gaps, workflow constraints, and variability in perceived value as central barriers to PRO implementation [19].

Opportunity constraints were prominent. Time limitations, competing priorities, and concerns about information burden shape whether tracking can be integrated into routine workflows. Previous implementation research in oncology has shown that monitoring systems are more likely to be adopted when embedded within existing clinical processes, supported by defined triage pathways, and aligned with established roles rather than added as an additional task [7-9]. EMR-integrated monitoring programs that include automated alerts and predefined response workflows have been described as feasible in ambulatory oncology settings when these structural supports are in place [20]. Observational analyses have also reported associations between participation in such programs and health care usage outcomes [8]. More broadly, implementation science literature emphasizes that long-term adoption of digital monitoring systems depends on minimizing disruption to clinical workflows and reducing cognitive and time burden on medical team participants [21], along with recent umbrella reviews highlighting workflow disruption, limited integration with EHRs, and time and resource constraints as key barriers to implementing patient-generated health data systems in oncology care [19]. The findings of this study add to this literature by identifying behavioral factors that may influence uptake of cardiovascular-specific tracking within cardio-oncology settings.

Motivational influences were equally apparent. Patients described reassurance and a sense of control as reasons for engaging in monitoring, whereas uncertainty about symptom meaning sometimes contributed to inconsistent reporting. Medical team participants were more inclined to engage with monitoring data when it was perceived as clinically meaningful but expressed concern when data volume was high or when response expectations were unclear. This aligns with previous real-world implementation studies demonstrating that long-term engagement with electronic PRO systems may vary over time and is influenced by reminder systems, medical team participant reinforcement, perceived relevance to care, and integration into clinical workflows [22]. These findings are also consistent with previous research showing that perceived usefulness and ease of use are central determinants of engagement with digital health and self-monitoring tools in chronic disease management [23]. These perspectives indicate that perceived usefulness and manageable data flow may be important for long-term engagement over time.

Taken together, these findings indicate that knowledge clarity, workflow integration, and perceived value operate in combination. Introducing a digital tool without addressing expectations or integrating tracking into existing care processes may offer limited benefit.

### Implications for Digital Health Design

Our findings inform the design of digital tools intended to support cardiovascular symptom tracking in cancer care. For patients, tools may benefit from providing clear guidance on which symptoms and signs to monitor and when changes should prompt communication with the medical team. Educational components embedded within the tracking process may help address gaps in psychological capability while avoiding additional burden during clinical encounters. Tools should also account for the overlap between cardiovascular, cancer-related, and treatment-related symptoms to avoid excessive data collection that increases patient burden or generates information that is not clinically actionable.

Medical team participants emphasized that digital tracking must be integrated into existing EMR systems and supported by clear triage processes. Previous implementation work in oncology indicates that feasibility is strengthened when monitoring systems include defined response workflows and designated roles for follow-up [20]. Broader implementation-oriented scholarship in digital oncology similarly emphasizes the importance of integrating patient-generated health data into existing clinical infrastructure and aligning digital tools with established workflows [21].

Broader mHealth research in populations with cancer has documented limited integration of patient-generated data into clinical systems and substantial variability in measures captured across platforms [9], echoing findings from recent reviews that emphasize the need for integrating patient-generated health data into clinical workflows and ensuring data are actionable at the point of care [19]. Participants emphasized that tracking is useful only when incoming information can be interpreted and acted upon efficiently. However, actionability may depend not only on data integration, but also on the availability of clinical resources, referral pathways, and support services that allow medical teams to respond to identified needs. For example, symptom or risk data may be more actionable in settings where patients can be referred to embedded supportive care, rehabilitation, behavioral health, nutrition, or cardiovascular services. In contrast, when follow-up services are unavailable, poorly coordinated, or associated with additional patient costs, tracking may generate information that is difficult to act on and may increase the burden for patients and medical team participants. This reflects broader challenges in translating patient-generated data into actionable clinical insights, particularly when data streams are not standardized or integrated with existing decision-support systems [9]. From an informatics perspective, this underscores the importance of systems that support interpretation within clinical workflows rather than treating data as isolated data entries [21].

In summary, digital cardiovascular symptom tracking systems should prioritize clarity, workflow alignment, and actionable outputs rather than simply increasing data volume. Participants’ perspectives also indicate that minimizing cognitive burden and time constraints for both patients and medical team participants may be central to feasibility. Clear expectations regarding symptom relevance and defined response processes may support more consistent engagement over time.

### Strengths and Limitations

This study has several strengths. First, it included perspectives from both patients and medical team participants, allowing examination of cardiovascular symptom tracking as an interdependent behavior rather than a unidirectional patient responsibility. Second, use of the COM-B framework provided a structured approach to organizing barriers and facilitators across psychological, social, and environmental domains while maintaining analytic transparency. Third, interviews focused specifically on cardiovascular symptoms and signs tracking in the cardio-oncology context, contributing to a growing body of research that has largely examined symptom monitoring more broadly in oncology.

Several limitations should be considered. Participants were recruited from a single, specialized cardio-oncology clinic and were predominantly highly educated and White, which may limit transferability to other institutions with different workflow structures or to more diverse and underserved populations. These findings may not reflect the experiences of individuals with lower health or digital literacy, limited access to technology, or those receiving care outside of specialized clinic settings. As with all qualitative studies, findings reflect self-reported perceptions and experiences rather than directly observed behaviors. We did not measure actual tracking adherence or clinical outcomes and therefore cannot draw conclusions regarding the effectiveness of specific tracking approaches. Additionally, because all interviews were conducted with participants who had access to videoconferencing capability, they may have been more interested in cardiovascular health or digital tools than nonparticipants, potentially introducing selection bias. Finally, while the COM-B framework provided a useful organizing structure, alternative theoretical approaches may have highlighted different aspects of tracking behavior. Future studies should include more diverse and community-based populations to better understand how cardiovascular symptom tracking can be implemented across different health care settings.

### Conclusions

Cardiovascular symptom tracking in cancer care is a shared behavior requiring coordinated engagement between patients and medical team participants. The use of the COM-B framework allowed identification of capability, opportunity, and motivation factors that shape how cardiovascular symptoms and signs are monitored and communicated in cardio-oncology settings. Participants described ambiguity regarding expectations across both groups. Patients emphasized uncertainty about which symptoms warranted tracking and about expectations for communicating symptom information, whereas medical team participants described time pressures, concerns about information burden, and limited standardized processes for reviewing and responding to symptom information. Medical team participants expressed concern about receiving large volumes of symptom information without clear mechanisms for prioritization or response, while patients described reassurance and a greater sense of control as benefits of tracking.

Efforts to strengthen cardiovascular symptom tracking may benefit from addressing behavioral determinants alongside technological considerations. Digital systems that provide clear guidance, align with clinical workflows, and support actionable interpretation of symptom reports may facilitate engagement and perceived value. Future studies should examine how these behavioral insights inform implementation strategies and evaluation of cardiovascular monitoring approaches in routine cancer care.

## Supplementary material

10.2196/100279 Multimedia Appendix 1 Patient and medical team interview guides.

10.2196/100279 Multimedia Appendix 2 Illustrative quotes for each theme from patients and medical team members.

## Abbreviations

COM-B: capability, opportunity, motivation–behavior
CVD: cardiovascular disease
EHR: electronic health record
EMR: electronic medical record
HRQoL: health-related quality of life
mHealth: mobile health
PRO: patient-reported outcome
PRO-TECT: patient-reported outcomes to enhance cancer treatment
RQA: rapid qualitative analysis

## References

[1] Ng HS, Meng R, Marin TS (2024). Cardiovascular mortality in people with cancer compared to the general population: a systematic review and meta-analysis. Cancer Med.

[2] Sturgeon KM, Deng L, Bluethmann SM (2019). A population-based study of cardiovascular disease mortality risk in US cancer patients. Eur Heart J.

[3] Wilcox NS, Amit U, Reibel JB, Berlin E, Howell K, Ky B (2024). Cardiovascular disease and cancer: shared risk factors and mechanisms. Nat Rev Cardiol.

[4] Liu S, Horowitz JD, Koczwara B, Sverdlov AL, Packer N, Clark RA (2024). Cardiac events among a cohort of 17,389 patients receiving cancer chemotherapy: short and long term implications. Cardiooncology.

[5] Paterson DI, Wiebe N, Cheung WY (2022). Incident cardiovascular disease among adults with cancer: a population-based cohort study. JACC CardioOncol.

[6] Bonsu JM, Guha A, Charles L (2020). Reporting of cardiovascular events in clinical trials supporting FDA approval of contemporary cancer therapies. J Am Coll Cardiol.

[7] Basch E, Schrag D, Jansen J (2025). Symptom monitoring with electronic patient-reported outcomes during cancer treatment: final results of the PRO-TECT cluster-randomized trial. Nat Med.

[8] Natori A, Sookdeo VD, Koru-Sengul T (2023). Symptoms and Needs Monitoring in Diverse Ambulatory Oncology Patients: Usage Characteristics and Impact on Emergency Room Visits and Hospitalization.

[9] Benzo RM, Gogineni A, Tetrick MK (2025). mHealth technologies in research studying cardiovascular health in cancer: a systematic review. PLOS Digit Health.

[10] Gregory ME, Cao W, Rahurkar S (2023). Exploring the incorporation of a novel cardiotoxicity mobile health app into care of patients with cancer: qualitative study of patient and provider perspectives. JMIR Cancer.

[11] Michie S, van Stralen MM, West R (2011). The behaviour change wheel: a new method for characterising and designing behaviour change interventions. Implementation Sci.

[12] Lyon AR, López-Fernández T, Couch LS (2022). 2022 ESC Guidelines on cardio-oncology developed in collaboration with the European Hematology Association (EHA), the European Society for Therapeutic Radiology and Oncology (ESTRO) and the International Cardio-Oncology Society (IC-OS). Eur Heart J.

[13] Hamilton A Qualitative methods in rapid turn-around health services research. Better evaluation knowledge.

[14] Hamilton AB, Finley EP (2019). Qualitative methods in implementation research: an introduction. Psychiatry Res.

[15] St George SM, Harkness AR, Rodriguez-Diaz CE, Weinstein ER, Pavia V, Hamilton AB (2023). Applying rapid qualitative analysis for health equity: lessons learned using “EARS” with Latino communities. Int J Qual Methods.

[16] Aanes SG, Wiig S, Nieder C, Haukland EC (2024). Implementing digital patient-reported outcomes in routine cancer care: barriers and facilitators. ESMO Real World Data Digit Oncol.

[17] Cresswell KM, Bates DW, Sheikh A (2013). Ten key considerations for the successful implementation and adoption of large-scale health information technology. J Am Med Inform Assoc.

[18] Greenhalgh T, Wherton J, Papoutsi C (2017). Beyond adoption: a new framework for theorizing and evaluating nonadoption, abandonment, and challenges to the scale-up, spread, and sustainability of health and care technologies. J Med Internet Res.

[19] Rammant E, Ramsey I, M de Ligt K (2026). Barriers and facilitators for implementing patient-reported outcome measures in oncology practices: an umbrella review. Qual Life Res.

[20] Penedo FJ, Medina HN, Moreno PI (2022). Implementation and feasibility of an electronic health record-integrated patient-reported outcomes symptom and needs monitoring pilot in ambulatory oncology. JCO Oncol Pract.

[21] Benzo RM, Noriega Esquives BS, Penedo FJ, Schneiderman N, Antoni MH, Penedo FJ APA Handbook of Health Psychology, Volume 2: Clinical Interventions and Disease Management in Health Psychology.

[22] Patt D, Wilfong L, Hudson KE (2021). Implementation of electronic patient-reported outcomes for symptom monitoring in a large multisite community oncology practice: dancing the Texas two-step through a pandemic. JCO Clin Cancer Inform.

[23] Tao D, Wang T, Wang T, Zhang T, Zhang X, Qu X (2020). A systematic review and meta-analysis of user acceptance of consumer-oriented health information technologies. Comput Human Behav.

