# Supplementary material for: Cardiovascular Symptom Tracking Among Patients With Cancer in Cardio-Oncology Care: Qualitative Study Using the Capability, Opportunity, Motivation–Behavior (COM-B) Framework
Source: JMIR Cancer. 2026 Jul 23;12:e100279. doi: 10.2196/100279 (PMC13395430; doi:10.2196/100279)
Supplement: Multimedia Appendix 1 [file cancer-v12-e100279-s001.pdf]

THE OHIO STATE UNIVERSITY

The Lifestyle Lab PI: Roberto Benzo Ph.D.
Division of Cancer Prevention and Control
1590 North High Street, Columbus, OH 43201

CVONC - Aim 1 Patient Interview Guide

Background

Thank you for agreeing to this interview. I am (NAME) from Ohio State University. We have invited you to participate because you have received cancer treatment that increases your risk for cardiovascular disease.

For this reason, we want to explore how an app could help you and your medical team track and manage your cardiovascular health to prevent disease. Tracking signs and symptoms help your medical team manage your care and can help inform patient-provider appointments.

There is currently no set way for your medical team to track and manage your cardiovascular health outside of the clinic, and often, signs and symptoms of cardiovascular health in cancer patients are missed. Because of this, we are developing a mobile application to help survivors such as yourself:

1. Track your signs and symptoms to manage your cardiovascular health
   a. **Symptoms are self-reported** and include things like fatigue, chest pain, or palpitations.
   b. On the other hand, **signs are often derived from devices** like blood pressure, weight, and activity levels.
2. Track and improve your physical activity to improve your cardiovascular health

During this interview, we want to hear your thoughts on these topics and how data from such an app and a physical activity intervention could assist you in managing your cardiovascular health. Of course, there are no right or wrong answers. It also means you are more than welcome to tell us if there is something that needs to be improved.

Voluntary Participation & Confidentiality

- The interview will take about 60-90 minutes. Before we begin the discussion, I want to ensure you understand that:
  - Your participation is completely voluntary. You can end the interview anytime. Your decision does not impact the care and treatment you receive.
  - The interview is confidential, meaning that your name will not be used in any reports or articles.
  - We will record the interview to collect data for our research. The recording will be transcribed and stored in a secured file on the OSUWMC network. It will not be used to identify you.
- Do you have any questions?

Demographic Questions

- First, may I ask your age, your gender, and your race and ethnicity?
- Could you please tell me a bit about yourself?

Clinical Details

1. Are you in active treatment, or have you already completed your treatment?
2. What type of cancer were you diagnosed with? When were you first diagnosed?
3. Have you or are you currently receiving treatment for your cancer?
4. When choosing a treatment option, did you learn of any potential risks to the heart associated with this treatment?

The Lifestyle Lab PI: Roberto Benzo Ph.D.
Division of Cancer Prevention and Control
1590 North High Street, Columbus, OH 43201

CVONC - Aim 1 Patient Interview Guide

Tracking Symptoms & Risk Factors

READ: Motivation refers to the internal factors influencing our decisions and behaviors, including our goals, habits, and emotions.

- *Can you describe the reasons for tracking your symptoms or signs (e.g., blood pressure)?*
  Symptoms are self-reported *and include things like fatigue, chest pain, or palpitations.*
  Signs are often derived from devices *like blood pressure, weight, and activity levels.*
  - *Can you describe how this might change before, during, or after treatment?*
- *Can you describe why you may not want to track your symptoms and signs?*
- *How could this application motivate you to track your symptoms and signs?*

READ: Opportunities refer to things outside of you that make tracking your symptoms or signs possible.

These can be physical opportunities, like time, things, or places. Or social opportunities like family, friends, or your medical team.

- *Can you describe any opportunities that help you track your symptoms and signs?*
  - *In other words, are there any external factors (time, places, or tools) that help you track your symptoms and signs?*
- *Can you describe opportunities that make it hard to track your symptoms and signs?*
  - *In other words, are there any external factors (time, place, or tools) that make it hard for you to track your symptoms and signs?*
- *Can you describe how an app could help you track your symptoms and signs?*

READ: The next questions are about your ability to track your symptoms and signs (e.g., blood pressure).

When we think of ability, we can think of physical abilities, like strength or stamina, or mental abilities, like knowing how to or remembering to do something.

- *How do you currently keep track of your symptoms and signs? (e.g., journaling, using an app, mental notes)*
- *When you want to track your symptoms and signs, are there techniques that you find helpful?*
- *Have you encountered any challenges or barriers while tracking your symptoms and signs? If so, how have you overcome them?*
- *How could this application help you better track your symptoms and signs?*

Experience with prior apps

CVONC - Aim 1 Patient Interview Guide

- **Can you tell me about any apps or online resources that help you track your symptoms and signs (e.g., blood pressure, weight, heart rhythm, activity)?**
-
    - **Probe:** Can you tell me about any app(s) or features included in that app that you **enjoyed**?
    - **Probe:** Can you tell me about any app(s) or features included in that app that you **disliked**?

- **Can you tell me about any apps or online resources that help you exercise or increase your activity?**
    - **Probe:** Can you tell me about any app(s) or features included in that app that you **enjoyed**?
    - **Probe:** Can you tell me about any app(s) or features included in that app that you **disliked**?

Features *(if time permits)*

Below is a list of app features identified through previous research. You are the expert, so we want your thoughts on the most important features we could include in an app to help you track your symptoms and increase your activity levels. Please rank the following features from most to least important and explain why (in-person, use note cards; if virtual, use Miro).

1. Ability to track symptoms occurrence.
2. Tracking specific symptom attributes (frequency, intensity, type, time)
3. Seeing trends in symptoms: Weekly, monthly, yearly.
4. Use the app or downloadable report to communicate symptoms with the medical team.
5. Access a community of other patients/survivors in similar situations (i.e., cancer patients at increased risk for cardiotoxicity).
6. Educational resources (e.g., education on symptom management, risk reduction strategies like Life's Essential 8, etc.)
7. A physical activity program to improve cardiovascular health

Cardiotoxic Symptoms *(if time permits)*

We are including the capability to track the following cardiotoxic symptoms in our app. Please rank the following **symptoms** from the most important to the least from your perspective:

1. Chest pain/tightness
2. Shortness of breath
3. Heart palpitations
4. Abnormal heart rate
5. Abnormal (high/low) blood pressure
6. Edema
7. Lightheadedness
8. Syncope
9. Excessive fatigue
10. Other: _______

Educational Resources *(if time permits)*

[Interviewer Script] We are considering including educational resources in our app. What information would be most helpful if the app included educational content?

Please rank the following **educational resources** from the most important to the least:

The Lifestyle Lab PI: Roberto Benzo Ph.D.
Division of Cancer Prevention and Control
1590 North High Street, Columbus, OH 43201

CVONC - Aim 1 Patient Interview Guide

1. Exercise program to improve cardiovascular health and reduce symptom burden.
2. Descriptions of symptoms, treatments, and risks.
3. Tips to improve cardiovascular health (e.g., life's essential 8).
4. Understanding of how cancer works and how the cardiovascular system or heart functions.
5. Other: ____________________

Wrap Up

Thank you for your participation! You will be receiving a digital gift card in your inbox.

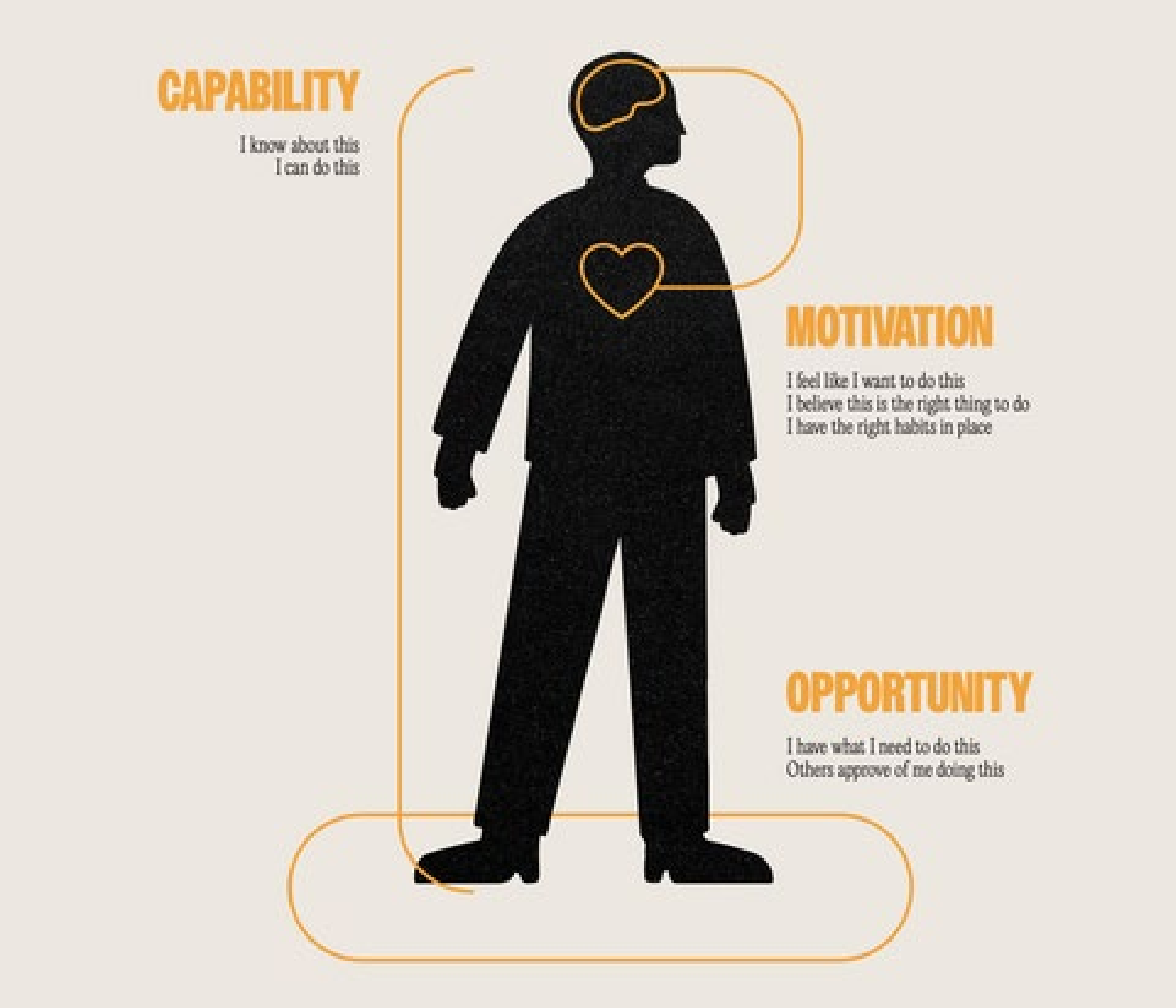

https://togetheragency.co.uk/news/the-com-b-model-of-behaviour-change-explained

THE OHIO STATE UNIVERSITY

The Lifestyle Lab PI: Roberto Benzo Ph.D.
Division of Cancer Prevention and Control
1590 North High Street, Columbus, OH 43201

Aim 1 - SME Interview Guide

Background

Thank you for agreeing to participate in this interview. I am (NAME), and I am from The Ohio State University.

We are developing a mobile application for cancer patients to 1) track of cardiotoxic symptoms and cardiovascular risk factors, and 2) promote physical activity. During this interview, we want to hear your thoughts on these topics, and how data from such an app and a physical activity intervention could assist you in caring for these patients.

The patients we are talking about include adults with a cancer diagnosis who are or have undergone a cancer regimen that includes a target, biologic, radiation, and immune-based therapy (e.g., TKIs and /or ICIs).

The **American Heart Association** has put together **Life's Essential 8**, the **key health behaviors and factors related to cardiovascular health.**

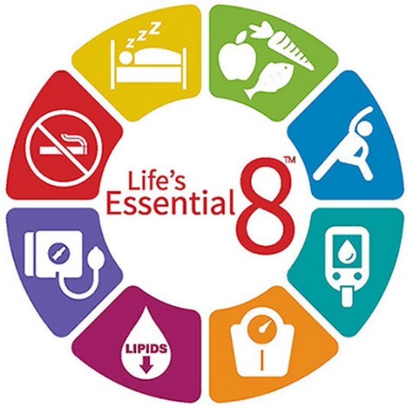

- Better cardiovascular health helps lower the risk of heart disease, stroke, and other major health problems in which you may be at risk due to cancer-related treatments.
- Regularly engaging in physical activity, one of the eight recommendations, can positively affect the many of these cardiovascular risk reduction factors (smoking, weight, cholesterol, blood sugar, blood pressure, sleep).

For this reason, we want to explore the idea of coupling this app with a **physical activity program** to help cancer patients' reduce their risk for cardiovascular disease.

Voluntary Participation & Confidentiality

- The interview will take about 60-90 minutes. Before we begin the discussion, I want to ensure you understand that:
    - Your participation is completely voluntary. If you choose to talk with me, you may end the interview anytime. Your decision does not impact in any way your job or employment status.
    - The interview is confidential. Your name will not be used in any reports or articles.
    - We will record the interview for data collection for our research. The recording will be transcribed and stored in a secured file on the OSUWMC network. The recordings will not be used to identify you in any way.
- Do you have any questions about this interview process?

Interview Questions

1. Can you state your name, current role, and how long you have been in this role?
2. Can you also tell us your age, your gender, and your race and ethnicity?

The Lifestyle Lab PI: Roberto Benzo Ph.D.
Division of Cancer Prevention and Control
1590 North High Street, Columbus, OH 43201

Aim 1 - SME Interview Guide

Job details

1. What type of cancer patients do you work with?
2. Tell us more about the role that cardiotoxicity plays when considering treatment options for cancer patients.

COM-B Self-Evaluation Questionnaire

The following checklist aims to identify areas where we may focus our discussion today to identify ways we could help patients track cardiotoxic symptoms and engage in a physical activity program to improve cardiovascular health. Please check or tell me which you believe impacts your ability to do each behavior (track cardiotoxicity symptoms and engage in regular physical activity). Some of these may sound strange or not applicable, but please focus on the ones that apply to you.

**Capability**			
In order to [i.e. regularly exercise], I would have to…	**Tracking Symptoms and Cardiovascular Risk Factors**	**Engaging in Regular Physical Activity**	**Examples**
Know more about why it was important			e.g., have a better understanding of the benefits of physical activity
Know more about how to do it			e.g., have a better understanding of effective ways to regularly exercise
Have better physical skills			e.g., learn how to regularly engage in activity
Have better mental skills			e.g., develop more confidence to exercise regularly
Have more physical strength			e.g., build up muscles to be able to exercise regularly
Have more mental strength			e.g., develop stronger resistance to not being active
Overcome physical limitations			e.g., come up with solutions to any activity restraints
Overcome mental obstacles			e.g., reduce unwanted urges or feelings
Have more physical stamina			e.g., develop a greater capacity to maintain physical effort
Have more mental stamina			e.g., develop a greater capacity to maintain mental effort
**Opportunity**			
	**Tracking Symptoms and Cardiovascular Risk Factors**	**Engaging in Regular Physical Activity**	**Examples**
Have more time to do it<br><br>**Physical Opportunity: Environmental Context and Resources**			e.g., create dedicated time during the day

The Lifestyle Lab PI: Roberto Benzo Ph.D.
Division of Cancer Prevention and Control
1590 North High Street, Columbus, OH 43201

Aim 1 - SME Interview Guide

Have more money<br><br>**Physical Opportunity: Environmental Context and Resources**			e.g., be given or earn funds to support regular exercise
Have the necessary materials<br><br>**Physical Opportunity: Environmental Context and Resources**			e.g., acquire better tools or resources to exercise regularly
Have it more easily accessible<br><br>**Physical Opportunity: Environmental Context and Resources**			e.g., provide easier access to facilities to exercise regularly
Have more people around them doing it<br><br>**Social Opportunity: Social Influences**			e.g., be part of a crowd/group of people also exercising
Have more triggers to prompt me<br><br>**Physical Opportunity: Environmental Context and Resources**			e.g., have more reminders at strategic times
Have more support from others<br><br>**Social Opportunity: Social Influences**			e.g., have one's family or friends' support
**Motivation**			
	**Tracking Symptoms**	**Engaging in Regular Physical Activity**	**Examples**
Feel that you want to do it enough<br><br>**Reflective Motivation: Intentions, Optimism**			e.g., feel more of a sense of pleasure or satisfaction from doing it (exercising)
Feel that you need to do it enough			e.g., care more about the negative consequences of not exercising regularly

Aim 1 - SME Interview Guide

**Reflective Motivation: Intentions, Optimism**			
Believe that it would be a good thing to do<br><br>**Reflective Motivation: Beliefs about Consequences, Optimism**			e.g., have a stronger sense that one should exercise regularly
Develop better plans for doing it<br><br>**Reflective Motivation: Intentions, Goals**			e.g., have a clearer and better developed plans for achieving it
Develop a habit of doing it<br><br>**Reflective Motivation: Intentions, Goals**			e.g., get into a pattern of doing it without having to think
Something else (please specify)			

**Symptom Tracking & Managing Cardiovascular Health**		
**COM-B Model Components**	**Theoretical Domain Framework (TDF) Domains**	**Interview Questions & Prompts**
Psychological Capability	Knowledge	• Can you tell me about cardiotoxic symptoms cancer patients experience?<br>○ **Prompt:** When do they occur, how often, their intensity?<br>• Can you tell me how cancer patients manage their cardiovascular health?<br>○ **Prompt:** Do they follow the guidance of the AHA Life's Essential 8?
	Memory, attention, and decision processes	• When was the last time you wanted to track a cancer patient's symptoms related to cardiotoxicity? How did you go about this?
Physical Capability	Physical Skills	• Can you describe in more detail how you keep track of your patient symptoms?<br>○ **Prompt:** How could this be improved or made useful?<br>• Can you describe in more detail how you help your patients' manage your cancer patients' cardiovascular health outside the clinic?<br>○ **Prompt:** How can you be confident that you understand your patient's cardiovascular health or life's essential 8 factors?

Aim 1 - SME Interview Guide

Social Opportunity	Social Influences	• Is there anyone to help track patient symptoms?<br>○ **Prompt:** Who could be of help and how?
Physical Opportunity	Environmental context and resources	• Beyond people, is there anything in the environment that could **help or hinder** you in tracking patient symptoms?<br>○ **Prompt:** Here are some examples, lack of time, using a journal, or an application
Reflective Motivation	Optimism	• (Ask if Time Permits) Are you confident that any barriers you may have to tracking patient symptoms can be solved?
	Intentions	• (Ask if Time Permits) Do you intend to keep track of your patients' symptoms?<br>○ **Prompt:** How so?<br>• (Ask if Time Permits) Do you intend to keep track of your patients' cardiovascular health risk factors (Life's Essential 8)?
	Beliefs about Consequences	• What do you think will happen if patients use an app to track patient symptoms? What about to track health behaviors and factors in Life's Essential 8?<br>○ **Prompt:** Discuss any benefits or consequences to track patient symptoms using an app?
	Goals	• What is your goal for your patients in terms of:<br>- *Symptom tracking*<br>- *Physical activity*<br>- *Cardiovascular health management*

Let's talk about the apps you use or have used and your experience.

Developing the app

1. What features would be most helpful if patients were to use an app to help them understand and manage their cardiovascular health?
   a. Prompts: Tracking vitals, symptoms, education, others?

[Note: If the participant does not like apps or is not savvy] I understand you may be hesitant to use apps to track patient health, but let's focus on the information and features you would find helpful in an app.

Our previous work explored developing an app to track cardiotoxic symptoms in cancer patients. Below is a list of features identified through previous research, but you are the expert, so we would like to get your thoughts on which, in your opinion, are the most important. Please rank the following and explain why (in-person use note cards; if the interview is held virtually, we will use Miro).

Features

[Interviewer Script] We are considering including the following feature in our app. Please rank the following **features** from the most important to the least:

1. Ability to track symptoms occurrence.
2. Tracking specific symptom attributes (frequency, intensity, type, time)

3. Seeing trends in symptoms: Weekly, monthly, yearly.
4. Use the app or downloadable report to communicate symptoms with the medical team.
5. Access a community of other patients/survivors in similar situations (i.e., cancer patients at increased risk for cardiotoxicity).
6. Educational resources (e.g., education on symptom management, risk reduction strategies like Life's Essential 8, etc.)
7. Physical activity program to improve health and reduce symptom burden.

Cardiotoxic Symptoms

[Interviewer Script] We are including the capability to track the following cardiotoxic symptoms in our app. Please rank the following **symptoms** from the most important to the least according to you as a healthcare professional.:

1. Chest pain/tightness
2. Shortness of breath
3. Heart palpitations
4. Abnormal heart rate
5. Abnormal heart pressure
6. Edema
7. Lightheadedness
8. Syncope
9. Excessive fatigue
10. Other: ______

Follow-up Question: *Would the ranking be any different from the patient's point of view?*

Educational Resources

[Interviewer Script] We are considering including educational resources in our app. If the app could display educational content in the app, what information do you think would be most helpful for the patient?

Please rank the following **educational resources** from the most important to the least:

1. Descriptions of symptoms, treatments, and risks.
2. Understanding of how cancer works and how the cardiovascular system or heart functions.
3. Tips to improve cardiovascular health (e.g., life's essential 8).
4. Exercise program to improve cardiovascular health and reduce symptom burden.
5. Other: ____________________

Aim 1 - SME Interview Guide

Example of a Miro Board Used for Ranking of Symptoms, Features, and Education Resources

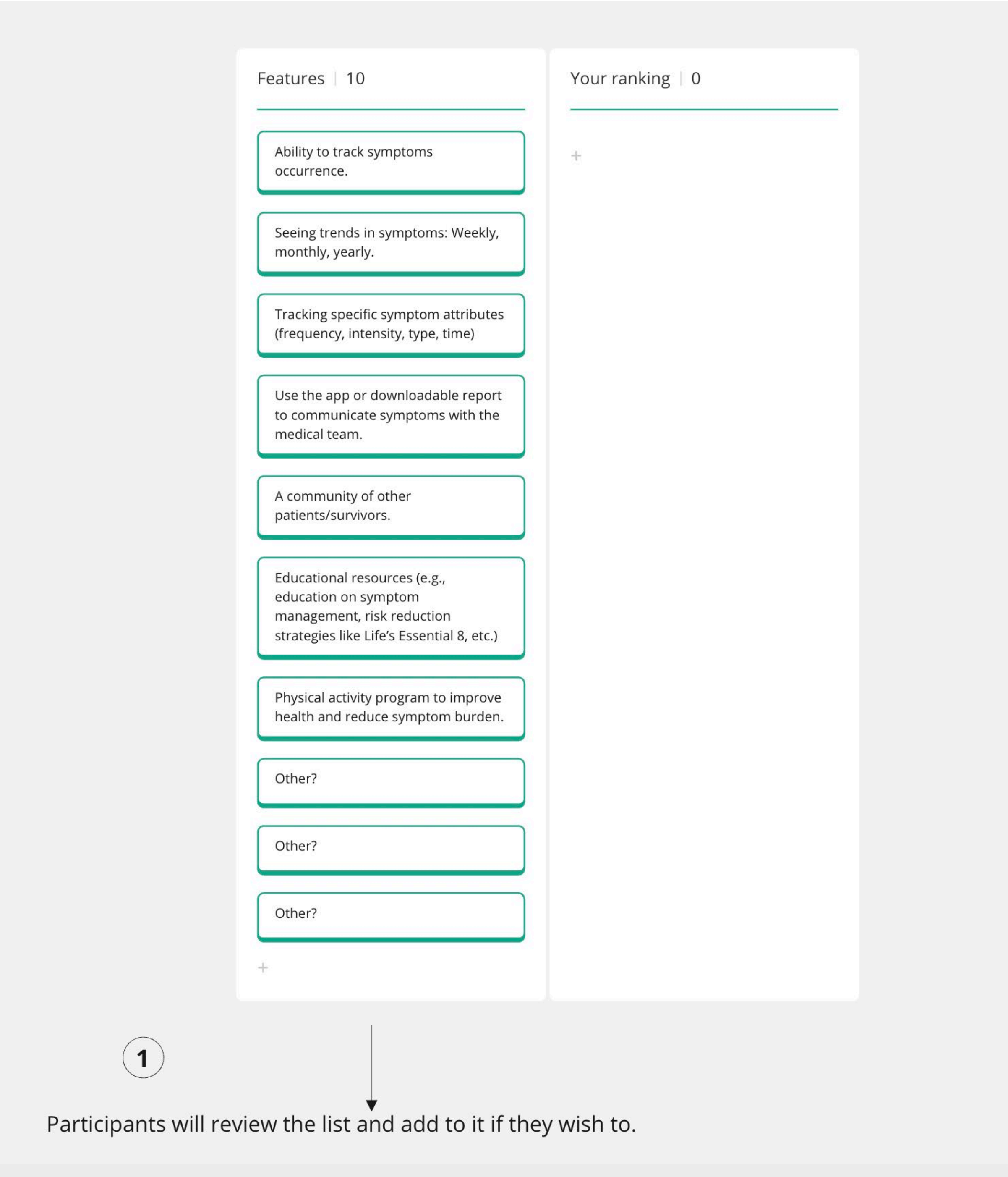

Caption: *Image 1 – Step one: Explanation of the Miro board for features, symptoms, and educational resources activities.*

Aim 1 - SME Interview Guide

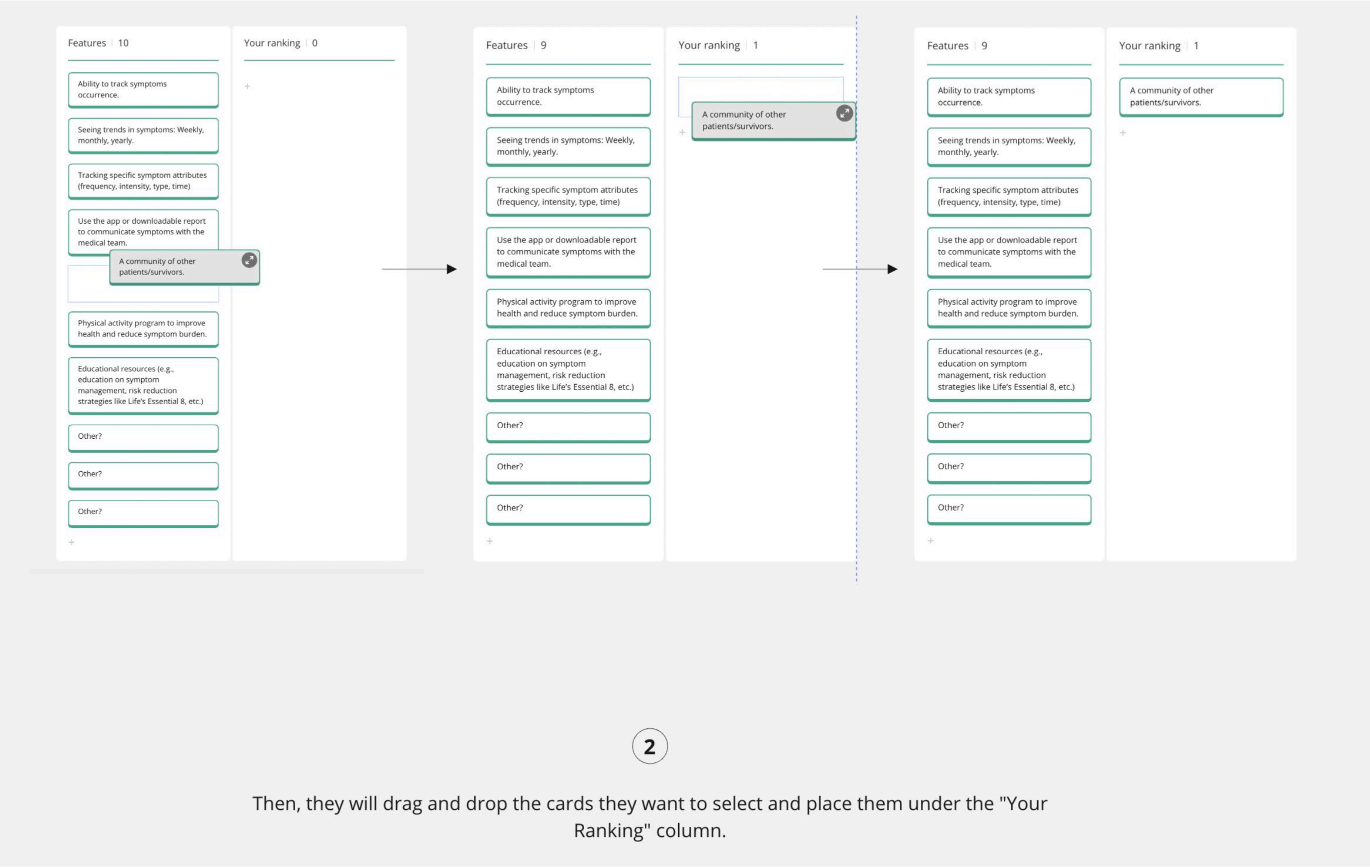

Caption: Image 2 – Step two: Explanation of the Miro board for features, symptoms, and educational resources activities.

Aim 1 - SME Interview Guide

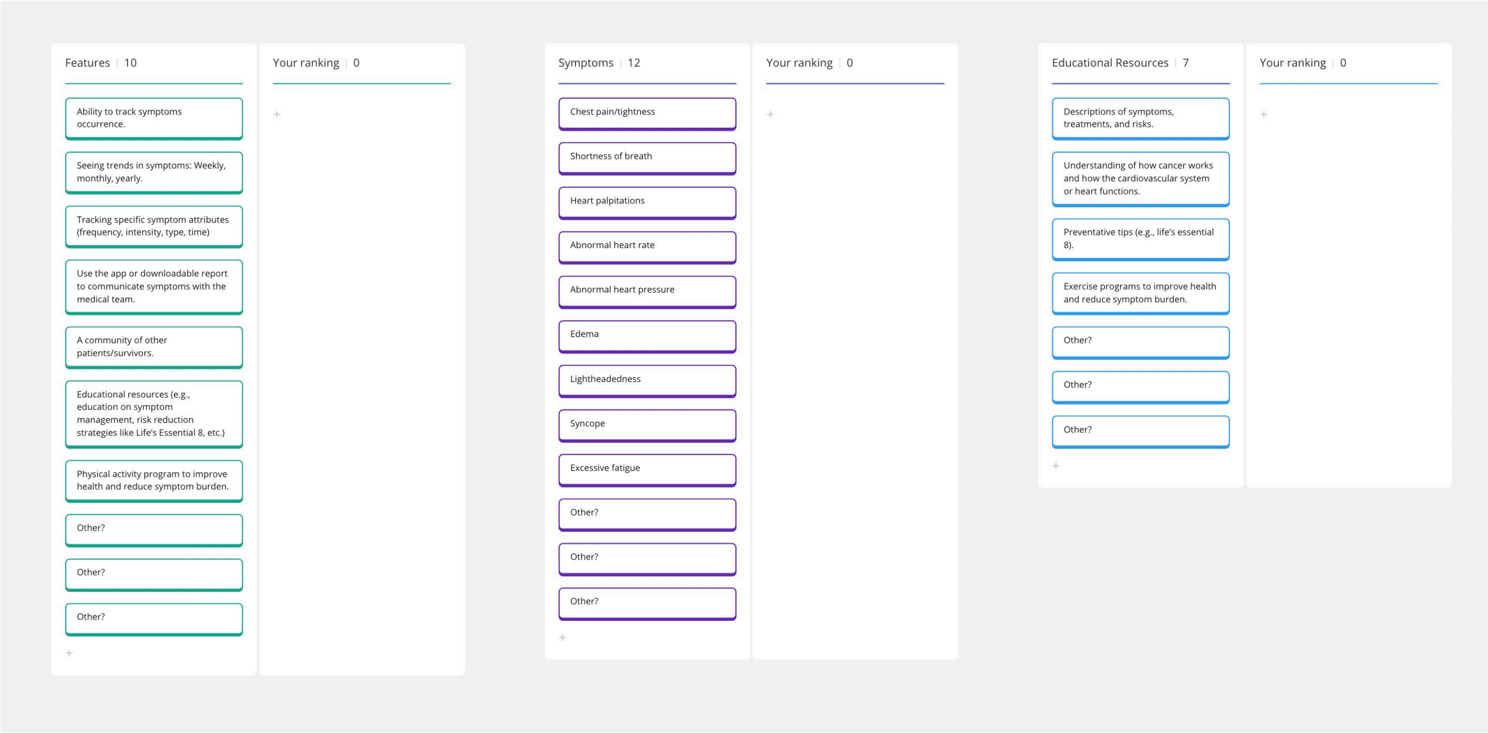

Caption: Image 3 - Features, symptoms, and educational resources activities on Miro board.

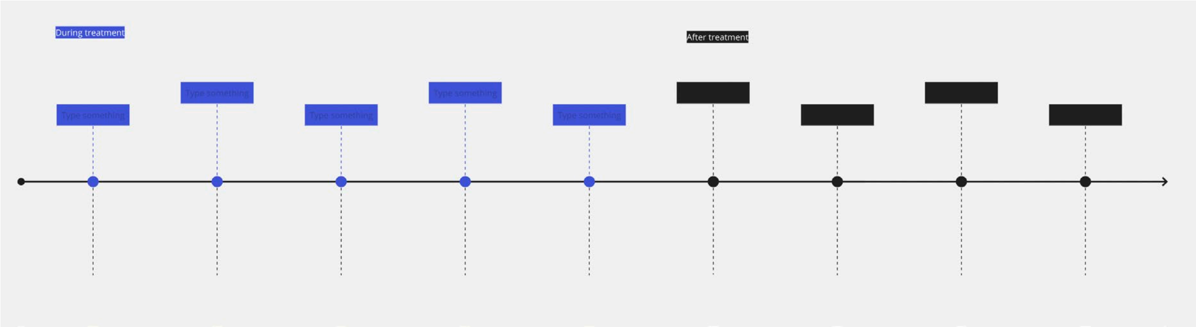

Aim 1 - SME Interview Guide

Reflective Motivation	Social/Professional Role	• (Ask if Time Permits) To what extent would tracking patient symptoms using an app be accepted by your colleagues or institution?
	Beliefs about capabilities	• What challenges or issues do you anticipate in patients accessing or using an app like this to track their symptoms?<br>○ **Prompt:** What are the **barriers** to tracking patient symptoms using an app?<br>○ **Prompt:** What are the **facilitators** for tracking patient symptoms using an app?<br>What challenges or issues do you anticipate in cancer patients participating in a digital physical activity program?

Automatic Motivation	Reinforcement	• (Ask if Time Permits) What incentivizes you to track patient symptoms using an app? What about to promote cardiovascular health?
	Emotion	• (Ask if Time Permits) Discuss how you think tracking patient symptoms to manage their cardiovascular health would make you feel?<br>○ **Prompt:** Would you feel empowered? Happy?

Communicating symptoms with Medical Team

1. What information about symptom burden do you rely on your patient to tell you?
2. What would it look like if the app could create a report of the patient's cardiotoxic symptom burden and cardiovascular health (Life's Essential 8) for the medical team?
    a. **Prompt:** What pieces of information would you like to see?
    b. **Prompt:** How could this help with clinical care? How could it inform patient visits?
    c. **Prompt:** What would make it useful?
    d. **Prompt:** How would you like to receive this report? (e.g., paper, email, EPIC in-basket)

Wrap Up

- Thank you for your participation!

The Lifestyle Lab PI: Roberto Benzo Ph.D.
Division of Cancer Prevention and Control
1590 North High Street, Columbus, OH 43201

Aim 1 - SME Interview Guide

If time permits, proceed with these questions.

EHR-Related Questions

1. What information do you typically rely on in your electronic health record (EHR)? What information is not there that you wish was?
2. If the app could push data to the patient's EHR, would this be helpful to you? What pieces of information (e.g., symptoms, etc.) would you like to see (please give us your top 5)? Please be as specific as possible.
