# Supplementary material for: Cardiovascular Symptom Tracking Among Patients With Cancer in Cardio-Oncology Care: Qualitative Study Using the Capability, Opportunity, Motivation–Behavior (COM-B) Framework
Source: JMIR Cancer. 2026 Jul 23;12:e100279. doi: 10.2196/100279 (PMC13395430; doi:10.2196/100279)
Supplement: Multimedia Appendix 2 [file cancer-v12-e100279-s002.docx]

| **Domain** |  | **Theme** | **# of Participants**  ***PC: Person with Cancer***  ***MT: Medical Team*** | **Quote** | **Quote** | **Quote** |
| --- | --- | --- | --- | --- | --- | --- |
| **Psychological Capability** | **PC Facilitators** | **Awareness/Knowledge of own symptoms and health** | 12 (PC 1-12) | *PC8: At one point my blood pressure was acting weird, you know, like, sometimes I get really low, or you know, or whatever the case may be, or sometimes it'd be much higher than normal.* | *PC11: So let's say I have headaches 3 days in a row. I think that's pretty weird, right? But having had brain surgery, it's not so weird. So I pretty much write them down, and I also keep track of weight and blood pressure on and off.* | *PC4: If something exists, it can be measured. So, you want to have some data collection. You don't*  *wanna go into your doctor and start explaining in really vague subjective terms, you wanna say, “My average is this, the medium is that,” you know, you wanna have objective data.* |
|  |  | **Was made aware of cardiotoxicity risks by medical team and asked to track** | 7 (PC 1, 2, 4, 7, 9, 10, 11) | *PC4: ...I knew that chemo was really hard on you, that it was a poison...so I was afraid of it, but I knew I had to do it, right? And then the transplant or, yeah, there's a lot of risks, but I knew that is something that I had to do. The alternative was death.* | *PC7: Yes, because when I was on the chemo, I did, it wasn't EKG,*  *what was it…a heart test, I can't remember. It's a common one where they do the sonar kind of thing, and I had to do that every couple of weeks, so he [provider] kept track of how my heart would respond.* |  |
|  | **MT Facilitators** | **Knowledge of cardiotoxicity** | 11 (MT 1-8, 10-12) | *MT5: All the patients I see in the ambulatory setting are cancer patients…So balancing their pre-existing cardiovascular disease, the presence or absence of pre-existing cardiovascular disease, and which cancer therapies they have received, or will be receiving, or are currently receiving are at the top of the list of considerations. So, the potential cardiotoxicity of cancer therapies is high on the list of consideration. And then, if there is already active cardiotoxicity and that's why they are referred to me, then it takes, it's number one reason, you know, to consider, you know, how we're going to manage things moving forward.* | *MT11: So really, the way that we tend to think about it, it's more based on the treatment, more than really the actual cancer disease itself. So, there are some cancers that tend to be treated more ubiquitously with certain types of drugs that, that are more, have a prolificity for cardiotoxicity... But anthracyclines are well associated with heart failure and a number of other cardiovascular issues.* |  |
|  | **PC Barriers** | **Did not know about cardiotoxic risks or how, when to track** | 5 (PC 5, 6, 8, 10, 12) | *PC8: I never really thought to ask about well, how will this affect my heart, or will this affect my heart in a bad way, or whatever.* | *PC6: No, we didn't discuss that. I don't remember. Well, I don't. I don't remember them discussing that, but I guess if I had looked at the papers that come with all the drugs that one drug that they took me off of definitely can impact the heart can be a side effect.* |  |
|  |  | **Recall Bias** | 5 (PC 1, 4, 5, 6, 7) | *PC5: You know, [oncologist name] has been asking me, and once again this is my recall. I could not be remembering correctly. But he, you know, he mainly asked, do you have chest pain? Do you have this? Do you have that? But I haven't been able to like, pull out a notebook and say, on February third I had this and February second.* | *PC7: You would say, oh, I'll mention this to a doctor next time, where you know. Sometimes you may think, wanting to mention it to the doctor, but then you go in and things happen, and then, of course, you forget to say anything, you know, and things like that.* | *PC1: Chemobrain, is very real, and it makes you pretty foggy, and I think, especially the days directly after treatment. I'm pretty foggy. So that's why, for me, like writing things down is an absolute must because otherwise it just doesn't happen.* |
|  |  | **Lack of tech-savviness** | 4 (PC 3, 7, 11, 12) | *PC3: Oh, you gotta, you gotta*  *keep in mind, I'm a bit*  *technologically challenged.* | *PC7: I'm not sure how this app thing will work, but I'm willing to try to make it work. And I'm not a real technical person.* |  |
|  | **MT Barriers** | **Hard to know which symptoms are cardiotoxic-related** | 8 (MT 1, 4-8, 11, 12) | *MT1: It can be heart failure symptoms like shortness of breath and swelling. They could have arrhythmias so they could have palpitations, dizziness, syncope. They could have like coronary artery disease symptoms so chest pain, heart attacks. They could have inflammation, so chest pain, shortness of breath, heart failure symptoms. And it can really be really mild, or it can be life-threatening so.* | *MT4: …the lightheadedness, syncope, I think those come up a lot in cancer patients. The issue with that is that it can be related to several different things, like, if patients are dehydrated, you know, not eating and drinking, it's gonna, it that's-, those are really difficult to attribute to, you know cardiac symptoms. But you know, a lot of, a lot of cancer patients experience those.* | *MT5: They're not always related to cardiotoxicity, it could just be coincidental that they have, that they're a cancer patient, but they also have heart disease. But they're not necessarily related to each other. But then, other times, it's clearly due to their cardiotoxicity of their cancer therapy. And other times, it's not clear, it could be, and we don't know definitively. So, you treat it either way.* |
| **Physical Capability** | **PC Facilitators** | **Journaling, using other recording tools to track** | 7 (PC 1, 5, 6, 8, 9, 10,11) | *PC11: I just mark, okay, I took these at this time. I took these at this time. I took these at this time so that I don't get mixed up.* | *PC6: Well, sometimes I write down so I don't forget to ask about or check on. I mean, I usually go with my little list. If I have questions, I mean, if I have questions or things happening between appointments or something, I write it down.* |  |
|  | **MT Facilitators** | **Provider asks about symptoms** | 12 (MT 1-12) | *MT1: Usually, I ask people to make a blood pressure log for me, and I tell them if they're having symptoms to write it down on the blood pressure log. So, I know what their blood pressure is, and then the symptom that prompted them to take the blood pressure. If it's that, or if they're just like monitoring their blood pressure for me.* | *MT7: I tend to ask my patients, those who have hypertension I always ask them to keep a log of blood pressure at home.* | *MT10: If somebody messages in, like, through their electronic medical record, through either the in-basket or like calling, calling in or sending in a MyChart message that’s really the only way that I hear about it.* |
|  | **PC Barriers** | **Symptoms Deter From Tracking** | 4 (PC 2, 4, 7, 8) | *PC2: It's about being comfortable and so for about 2 months we kind of fell off the wagon of meticulously tracking things. Once I started feeling better, it's sort of like, oh, we gotta get back together, get everything pulled back together.* | *PC7: I was on different drugs over the course of time. There would be side effects to certain ones that would be like feeling forgetfulness…* |  |
|  | **MT Barriers** | **N/A** |  |  |  |  |
| **Social Opportunity** | **PC Facilitators** | **Patient-Provider encounters** | 11 (PC 1-11) | *PC1: Every time I meet with my doctor, we have check ins and things, and you might get a good snapshot of, you know, what's happening on that morning when they ask you those questions. But being able to kind of understand my body's process of how it goes through all of the previous 3 weeks, and talk to them with any concerns or patterns that I see that they might not be aware of, I'm not aware of them, I think that's really important.* | *PC3: When I lost my wife, she and [provider’s name] were really close. Because [wife’s name] had open heart surgery at OSU about 2 and a half years ago, and she and Olivia came very close. And I remember the first time I went up to see her after my wife passed, she was so emotional we could hardly, we could hardly speak, she was just so kind.* | *PC7: I know a lot of times we go through, at the appointment, we would go through*  *what could be the side effects, or if I had any feeling*  *of those side effects, you know. Then, if there were too many of them, or if taking the medicine longer, they weren't going away, then we would change.* |
|  |  | **Social Support** | 8 (PC 1, 2, 4, 5, 6, 7, 11, 12) | *PC4: My husband and I had a worksheet. You know, we had a sheet, and you had to mark it off, and it wasn't 100% perfect, but you need the structure, and now I find I need a pill container as glamorous as that sounds. It's the only way I can really remember.* | *PC1: My husband has been great cause he wants me to feel good. So I think, having like a support system of of people that want you to feel as good as you can and are concerned when you don't feel good and kind of want to mark that, that's important.* | *PC12: My wife's got her doctorate … she's the one that can sit there and talk the doctors’ lingo… so*  *without her, though, my communication with my doctor that would have been dead years ago.* |
|  | **MT Facilitators** | **Patient-Provider encounters** | 7 (MT 1, 2, 4, 5, 7, 10, 12) | *MT4: Yeah, I mean every patient encounter we ask some of those questions, or some of, for those symptoms that we discussed. For example, swelling comes up a lot…shortness of breath comes up with almost every single patient encounter, and again, like I mentioned, it can be related to cardiac toxicities, or it could be related to something else.* | *MT10: …during treatment for radiation for like esophagus cancer, for example, it's a 5 and a half week long treatment. So, we see them every week. They, you know, they kind of report what's going on every week.* |  |
|  |  | **Social Support** | 7 (MT 1-5, 7, 11) | *MT5: Usually, the family members that come…usually you get a fair bit of involvement of family… But a lot of times it is kind of nice to have another level of engagement just because it either helps corroborate the story of what the patient is telling me or can help clarify. I mean, you know, patients run chemo and things. Sometimes they get a little forgetful.* | *MT7: They all have a caregiver, they need it… So, what the patient doesn't tell me the relative or the caregiver will….So I have patients that are very quiet and they don't tell much about, like, they may portray like everything is fine, when in reality they might have symptoms. So, usually the caregiver is the one who tells me.* | *MT2: So, their family and friends are huge in getting the full picture of the patients a lot of times.* |
|  |  | **Staff support** | 5 (MT 1, 5, 6, 11, 12) | *MT5: I don't have the bandwidth to call every patient and check on them and the patients I see here on Wednesday. [Nurse's name] helps with that.* | *MT6: We have had a nurse here who would look at those numbers once a week just to make sure the numbers are staying within the range, or would be going out of the range.* | *MT11: …some systems set things up where patients are allowed to talk to nurses, to almost help filter out information before they, you know, reach a physician. They'll let nursing staff or other staff, kind of help filter the information and then send it on to the clinician so that they can respond to those that are pertinent.* |
|  | **PC Barriers** | **Provider not requesting data** | 6 (PC 6, 7, 9, 10, 11, 12) | *PC6: No, I don't think they've ever asked. If I've they said it's*  *important. It's good if you can record this, but I don't think*  *anybody's ever asked if I recorded it or not.* | *PC9: ...they'll ask me about appetite and all those kind of things, but in terms of a quantitative monitoring I would say, not really.* |  |
|  | **MT Barriers** | **Limited staff support** | 2 (MT 5&9) | *MT9: ...we just don't have enough like time, or like staff, honestly to like, follow up with all of these patients routinely, you know.* |  |  |
| **Physical Opportunity** | **PC Facilitators** | **Physical or digital tools** | 11 (PC 1-11) | *PC1: I have a little like stationary that I keep some stuff on, especially with the heart stuff. There's like my blood pressure and things they've asked me to track. I also happen to just like journal most days. And so that's a pretty good way to remember.* | *PC3: Well, when I take my blood pressure right? If it's, if it's, out of range, I'll record it and the date that I do it on a calendar.* | *PC5: And so, I went back in my phone's calendar, looked up some things to help me. That was what dates I had certain symptoms, or what have you.* |
|  | **MT Facilitators** | **Physical or digital tools** | 10 (MT 1, 2, 3, 5, 6, 8-12) | *MT1: I have all of my books and resources and things. I ask the library lady on Tuesday mornings, who comes to East, if she has any resources for cardiovascular health. ...she gives me all these like huge stacks of stuff like I have blood pressure logs, I have 100 calorie snack list, I have cholesterol, I have like all this stuff that I can't carry it around with me. But yeah, I like to give, I like to give people physical things that they can look at when I can. So, blood pressure logs, yes, I go through those, I go through like a million.* | *MT2: "Sometimes they ask for printouts of, you know, what did my blood pressure look like from day one to day 36? And they'll be like, or, 'I'm going to see my doctor for a 6 month follow up. I've been in rehab for 2 months. Can you give me a printout of what my blood pressures look like, so that they kind of get an idea of what's going on?' So, I think it's really good for practitioners as well.* | *MT10: A lot of people do come in, and they, like, write things on paper, like…'here were my vitals for the last week.'* |
|  | **PC Barriers** | **Lack of time** | 6 (PC 1, 2, 4, 5, 8, 9) | *PC2: Time is very important. It's very big. Like my life right now is structured upon again, 9 AM to 9 PM. Kind of that's when I do meds and do shots and everything.* | *PC1: So I think cause sometimes, especially when I was working full time and also trying to like manage treatments and stuff, it just felt like there was such a limited amount of time and memory to do those things (tracking).* |  |
|  | **MT Barriers** | **Lack of physical or digital tools** | 10 (MT 1-5, 7, 8, 10, 11, 12) | *MT5: ...having the resources to be able to do it, right. Have a phone, do you have Wi-fi, you know, I mean, we take it for granted, but there's still lots of people that don't have the means to have a smartphone and, or Wi-fi in their house. So, that may be a limiting factor.*  *MT5: I was giving them like manual sheets of paper...they would keep a log, and then send us once a month the log. It was very cumbersome to keep up with all those patients and get them to actually send it.* | *MT4: I think what would be helpful is if somehow you can include patients’ history to kind of monitor how patients like, for example, if someone says, 'I had some chest discomfort', maybe they can click a button and say, this is when it started, this is when it ended, and that would save it on the record, and so that we know exactly how long it lasted.* | *MT10: I mean, I've often thought like how nice it would be to keep track of patients when they're not in my clinic to make sure that everything's going okay, and they're not in trouble, but then also for the patient to kind of log things and kind of see their progress and see whether, you know, how they've progressed and how they're recovering.* |
|  |  | **Lack of time** | 7 (MT 1, 5, 6, 9-12) | *MT5: I don't have the bandwidth to call every patient and check on them and the patients I see here on Wednesday…So you know, kind of calling in and checking either definitely on a daily basis, even on a weekly basis, can be challenging for every patient that you're curious about.* | *MT11: There's just a lot of things that you have to do like notes and orders, and all these other kind of things that are kind of responsibilities for clinicians. That's in addition to all the other things that you have to do, that you're engaged in.* |  |
| **Reflective Motivation** | **PC Facilitators** | **Perceived value of monitoring for health management** | 11  (PC1-11) | *PC8: The one thing that I enjoy is being able to look at the data. I mean, that's just my thing. I like being able to look and see my trends where I've been, where I'm going.* | *PC1: Honestly, I think that knowledge and data are kind of like a huge piece of mind for me personally like being to look able to look at trends especially like makes me think about chemo both with, you know, the signs of like my blood pressure, my heart rate, but also with the symptoms being able to kind of plan how I'm going to feel, I know on Day 5. I'm going to feel, you know, the most tired, and things like that. Allows me to kind of find the best way to live my life to the max, you know.* | *PC4: So you want to have some data collection you don't wanna go into your doctor and start explaining in really vague subjective terms, you wanna say. my average is this, the medium is that you know, you wanna have objective data.* |
|  |  | **Planning their daily lives** | 2  (PC 1 and 2) | *PC1: If I know when I'm going to be tired, I'm not going to make plans for those days. I'm going to try to give myself rest. But then, on the days where I know I start ticking up, then I know that I can start making plans with people, or we can choose to take a trip at that time.* | *PC2: At times you have to plan, we have found out, we gotta really plan around how we're going to do things. Versus in the old days, it was just like, ‘Oh, hey! Pack a bag! Let's go for the weekend’, you know?* |  |
|  | **MT Facilitators** | **Clinical decision making** | 10  (MT1-6, 9-12) | *MT5: But having better kind of updates on their symptom progression will allow us to better manage their cardiotoxicity or cardiovascular disease that may be independent from their cancer, cancer therapy.* | *MT11: …It [their heart] was very compromised by the time they came in. And to some extent, you wish that it had been captured earlier, because we'll, we'll try to do what we can now, but in some cases, these things don't improve. But we know with things like heart failure and other diseases, if you catch them early, you're able to intervene.* | *MT6: You ask them whether they're doing okay, whether they're having any symptoms. If they’re having any symptoms, then you try to make adjustments to the medications.* |
|  |  | **Understanding health outside of the clinic** | 3  (MT3, 4, 7) | *MT3: So…blood pressure monitoring is, I think, a very important thing to eliminate the confounding effects of what happens in the office, white coat syndrome, anxiety, or whatever happens in the office. So I highly believe in home readings.* | *MT4: Usually, I ask them, you know, 'When you go home over the next, you know, month or so before I see you next, I want you to write down your blood pressure at different times of the day and make sure that you bring that back. So I know, you know, is your blood pressure really high at home, or is it just high in clinic because you're anxious?'* |  |
|  |  | **Patient motivation and awareness** | 5  (MT1, 2, 6, 9, 12) | *MT6: Awareness is the first thing, because once there is awareness, then the motivation.* | *MT2: The big thing is for them to be self-aware of what's going on… So, being more self-sufficient and taking control of their health is the biggest goal, because, like so, we can't hold their hand forever.* | *MT9:…people will change when, like, they're ready to change, not necessarily when someone tells them to change. If they can come to the conclusion that, like, I want to do this and I can do it because I can connect the dots on how this will affect my health long term, I mean, ultimately, that's like what I think what creates sustainable long-term change.* |
|  | **PC Barriers** | **Lack of emphasis to track from providers** | 3  (PC 2, 6, 11) | *PC11: Breast cancer is the only cancer you get where you gain weight because of all the hormone issues and all that kind of stuff, and I would bring up the topic. And they would say, as long as you feel good, we're not worried about your weight. Well, I was worried about my weight.* | *PC6: Well, they just said you should watch your blood pressure, but I mean I do, but it doesn't make any difference, because when you go they just take it that day, and that's all.* | *PC6: The doctor said, “Get your weight down to a certain weight.” So, I worked at that for like 6 or 8 months, and then I went back for my checkup, they never commented or checked it, or said a thing about it. So what was the difference if I did or didn't?... I mean, you're not very motivated once you do something, and then nobody even comments about it.* |
|  |  | **Overwhelmed by demands of being a survivor** | 4  (PC 1, 4, 8, 12) | *PC1: I think it gets to be just a lot sometimes. Both logistically, in terms of like, there's so many appointments, there's so many medications. There are so many points, again, of like data, of like taking care of yourself, that sometimes you're just like, I want to sit on this couch all day. I don't want to think about any of this crap like I need a day or 2 or 3, you know.* | *PC4: I'd have to say when I'm first confronted with like information or diagnosis, or this or that, I can be extremely compliant in thinking I'm gonna track my blood pressure, you know, and do this, do that. And then a lot of times, you do it for a couple of weeks, and then it's like the January first resolutions.* |  |
|  | **MT Barriers** | **Don’t want overload of information** | 4 (MT 1, 3, 5, 10) | *MT10: You run the risk of collecting too much information, and, you know, kind of overburdening… You know, everybody's busy nowadays. And so, like sorting through, you know, like reams of paperwork is not necessarily gonna be super helpful.* | *MT5: And who's going through that? If you have a hundred patients… that are constantly, daily, giving you data about how much fluid they have, that's a full time job for someone to comb through all that data.* |  |
|  |  | **Patient motivation and burden** | 5 (MT 1, 3, 5, 8, 12) | *MT3: I don't know how expensive this device is, I know my patients are old, they're not tech savvy. And I don't know if I add this additional burden to their day-to-day thing, how they will embrace it.* | *MT8: I feel like it's fatigue and just appointment burnout in a lot of my patients. Their new identity is being a patient and so when they can spend time at home, the last thing they wanna do is more things that remind them of being a patient, and they'd rather spend time with their family or zone out and watch TV, or, you know, play video games, things like that, and try to feel some sense of normalcy.* |  |
| **Automatic Motivation** | **PC Facilitators** | **Habitual tracking** | 7 (PC 1-4, 7, 9, 11) | *PC7: So it's just kind of a routine. I guess it just kind of reassures me that I know what's right. I just feel like it's probably, you know, a lot psychological, but it feels like I'm keeping track of something, my body, you know, and I've got control of something right?* | *PC3: No, I'm kind of a creature of habit. You know, I know what I want to take, I know what I want to keep track of, and I do it.* | *PC9: I do have a monthly diary where I note when I take my medications.* |
|  |  | **Emotional processes** | 6 (PC 1, 2, 7, 8, 10, 11) | *PC2: If I'm kind of out of it like today, I'm kind of lethargic. may be an indication of something like bigger and that that's important to kind of note, especially if it occurs over a period of time.* | *PC2: If I don't feel good. If I start to feel really rough, I'll check my blood, level, my blood glucose levels and that would be the one where sometimes you can feel, oh, man! Still like really bad! And look at it.* | *PC8: And that affects me in different ways, emotionally, physically, all different ways, because it's like, “Oh, gosh! I gotta take all these medicines, you know, every day.” So, and at one point my doctor had me tracking my blood pressure…so I have a blood pressure*  *monitor here at home.* |
|  | **MT Facilitators** | **Habitual tracking** | 3 (MT 4, 5, 11) | *MT11: So, we ask the same questions. So, we ask even seemingly basic questions like every time…. We ask 5 to 6 questions in the cardiology practice, especially in pretty much most of the patients.* | *MT4: I mean every patient encounter we ask some of those questions, or some of, for those symptoms that we discussed.* |  |
|  | **PC Barriers** | **Emotional processes** | 6 (PC 1, 4, 6, 8, 9, 11) | *PC4: Sometimes I see myself as going overboard with tracking so much that you become too somatically focused, and it starts becoming anxiety-producing. Because you're always tuned into every breath, and this and that, rather than living your life and having fun and doing things that are fun.* | *PC9: That's one of the things I wanted to say that's frustrating to me, because I'd like to be able to get on there and say, “Hey, I felt some, a fair amount of stuff last night. Is that associated with an Afib episode or not?” And I cannot do that.* |  |
|  | **MT Barriers** | **N/A** |  |  |  |  |
